# Nuclear m^6^A reader YTHDC1 regulates alternative polyadenylation and splicing during mouse oocyte development

**DOI:** 10.1371/journal.pgen.1007412

**Published:** 2018-05-25

**Authors:** Seth D. Kasowitz, Jun Ma, Stephen J. Anderson, N. Adrian Leu, Yang Xu, Brian D. Gregory, Richard M. Schultz, P. Jeremy Wang

**Affiliations:** 1 Department of Biomedical Sciences, University of Pennsylvania, Philadelphia, United States of America; 2 Department of Biology, University of Pennsylvania, Philadelphia, United States of America; 3 Department of Anatomy, Physiology and Cell Biology, School of Veterinary Medicine, University of California, Davis, Davis, United States of America; University of Nevada School of Medicine, UNITED STATES

## Abstract

The *N*^6^-methyladenosine (m^6^A) modification is the most prevalent internal RNA modification in eukaryotes. The majority of m^6^A sites are found in the last exon and 3’ UTRs. Here we show that the nuclear m^6^A reader YTHDC1 is essential for embryo viability and germline development in mouse. Specifically, YTHDC1 is required for spermatogonial development in males and for oocyte growth and maturation in females; *Ythdc1*-deficient oocytes are blocked at the primary follicle stage. Strikingly, loss of YTHDC1 leads to extensive alternative polyadenylation in oocytes, altering 3’ UTR length. Furthermore, YTHDC1 deficiency causes massive alternative splicing defects in oocytes. The majority of splicing defects in mutant oocytes are rescued by introducing wild-type, but not m^6^A-binding-deficient, YTHDC1. YTHDC1 is associated with the pre-mRNA 3’ end processing factors CPSF6, SRSF3, and SRSF7. Thus, YTHDC1 plays a critical role in processing of pre-mRNA transcripts in the oocyte nucleus and may have similar non-redundant roles throughout fetal development.

## Author summary

The *N*^6^-methyladenosine (m^6^A) modification, one type of RNA methylation, is the most abundant internal RNA modification in eukaryote messenger RNAs. m^6^A is specifically recognized by RNA-binding reader proteins. Here we report an essential role of the nuclear m^6^A reader, YTHDC1, in embryo development and fertility. In particular, YTHDC1 is required for oocyte growth and maturation. YTHDC1-deficient oocytes exhibit massive defects in alternative splicing, which can be rescued by introducing into mutant oocytes wild-type, but not m^6^A-binding-deficient, YTHDC1. Strikingly, loss of YTHDC1 causes extensive alternative polyadenylation in oocytes, resulting in altered 3’ UTR length. YTHDC1 interacts with the pre-mRNA 3’end processing factors CPSF6, SRSF3, and SRSF7. Thus, YTHDC1 is a key nuclear factor in the processing of pre-mRNA transcripts.

## Introduction

More than one hundred different RNA modifications are known in eukaryotes [1]. *N*^6^-methyladenosine (m^6^A) is the most prevalent internal modification in eukaryote mRNAs, occurring in transcripts of approximately one third of genes in human and mouse [2–4]. Globally, m^6^A is enriched in the 3' most exons, long internal exons, and 5’ untranslated regions (UTRs) [5–9]. In addition to mRNAs, m^6^A is also present in long non-coding RNAs such as *Xist*, small nuclear RNAs, and ribosomal RNAs [10–12]. The m^6^A RNA modification is widely conserved among eukaryotes including yeast, flies, and plants [13–17].

Generation of m^6^A is catalyzed by a multi-component methyltransferase (m^6^A writer) consisting of methyltransferase-like 3 (METTL3), methyltransferase-like 14 (METTL14), and Wilm’s tumor associated protein (WTAP) [8, 18–20]. m^6^A is a reversible modification and two m^6^A demethylases have been identified: fat mass and obesity-associated protein (FTO) and alkB homolog 5 (ALKBH5) [21, 22]. Readers of the m^6^A mark preferentially bind to m^6^A and elicit downstream functions. Five mammalian m^6^A readers contain the YTH (YT521-B homology) domain: YTHDF1, 2, 3 and YTHDC1, 2 [6, 23–26]. YTHDF1, 2, and 3 are cytoplasmic [6, 23]. YTHDC1 localizes to the nucleus in cultured mammalian somatic cells [27, 28], whereas YTHDC2 is cytoplasmic in meiotic spermatocytes [29–33]. The m^6^A modification occurs preferentially at the conserved RRACH motif (R: G or A; H: A, C, or T) [34]. The YTH domain is an RNA-binding motif [35] and crystal structural studies reveal that the YTH domain of YTHDC1 selectively binds to m^6^A in the consensus motif [24, 25]. In addition to the five YTH domain-containing m^6^A readers, a number of RNA-binding proteins lacking a YTH domain are m^6^A readers: IGF2BP proteins [36], FMR1 [37], the translation initiation factor eIF3 complex [38], HNRNPA2B1 [39, 40], HNRNPC [41], and HNRNPG [42]. The HNRNP family members are considered “indirect” m^6^A readers, because m^6^A alters the local RNA structure to facilitate their binding to m^6^A [4, 40–42].

m^6^A functions in key RNA metabolic processes. m^6^A regulates gene expression [5, 6], mRNA stability [23, 43], translation efficiency [44, 45], alternative splicing [15, 16, 46], and cytoplasmic mRNA turnover [23, 47]. m^6^A is also involved in a number of developmental processes. In yeast, m^6^A formation occurs only during meiosis and is catalyzed by IME4, which is the sequence homologue of mammalian METTL3 and induces meiosis [13, 14]. m^6^A modulates alternative splicing of *Sxl* (sex lethal) transcript and thus sex determination in *Drosophila* [15, 16]. m^6^A is abundant on the long non-coding RNA *Xist* and promotes *Xist*-mediated gene silencing during X-inactivation [10]. Inactivation of *Mettl3* in mouse or *IME4* in *Drosophila* leads to embryonic lethality, demonstrating an essential role for m^6^A in lineage differentiation [48, 49]. Mouse *Mettl3* is required for spermatogonial development and spermatogenesis [50, 51]. Disruption of the m^6^A demethylase gene *Alkbh5* causes male infertility in mouse [22], whereas YTHDC2 is required for spermatogenesis and oogenesis in mouse [26, 31–33]. YTHDF2-mediated clearance of maternal transcripts promotes zygotic genome activation in zebrafish [52]. Mouse YTHDF2 regulates maternal transcript dosage and is essential for female fertility [53]. In addition, knockdown studies have uncovered a role of m^6^A in zebrafish development [20], circadian rhythm [54], cell reprogramming [7, 49, 55], and miRNA biogenesis and effects [39, 56]. Therefore, m^6^A plays important roles in a large number of developmental processes.

We previously identified YTHDC1 as a meiotic chromatin-associated protein in a proteomic screen [57]. YTHDC1 (initially referred to as YT521-B) changes alternative splicing patterns in a concentration-dependent manner [27] and localizes to nuclear speckles, which contain active transcription sites [28]. Tyrosine phosphorylation of YTHDC1 regulates its intra-nuclear localization, thereby modulating its effects on alternative splicing [58]. YTHDC1 promotes exon inclusion by recruitment of serine/arginine-rich (SR) splicing factor 3 (SRSF3), a pre-mRNA splicing factor [46]. YTHDC1 facilitates nuclear export of m^6^A-containing mRNAs through SRSF3 and NXF1 [59]. Although these studies in cultured cells have provided important insights into the function of YTHDC1, its requirement during development is unknown. In addition, the biological function of accumulation of m^6^A sites in 3’ UTRs remains mysterious. Here, we report that YTHDC1 is essential for embryonic development in the mouse. Using a conditional inactivation approach, we find that YTHDC1 is required for survival of spermatogonia in males and controls postnatal oocyte development in females. Strikingly, in addition to alternative splicing defects, loss of YTHDC1 causes widespread alternative polyadenylation in oocytes. Importantly, YTHDC1 is associated with SR proteins and pre-mRNA 3’ end processing factors.

## Results

### Nuclear localization of YTHDC1 in male germ cells, oocytes and pre-implantation embryos

**Fig 1:**
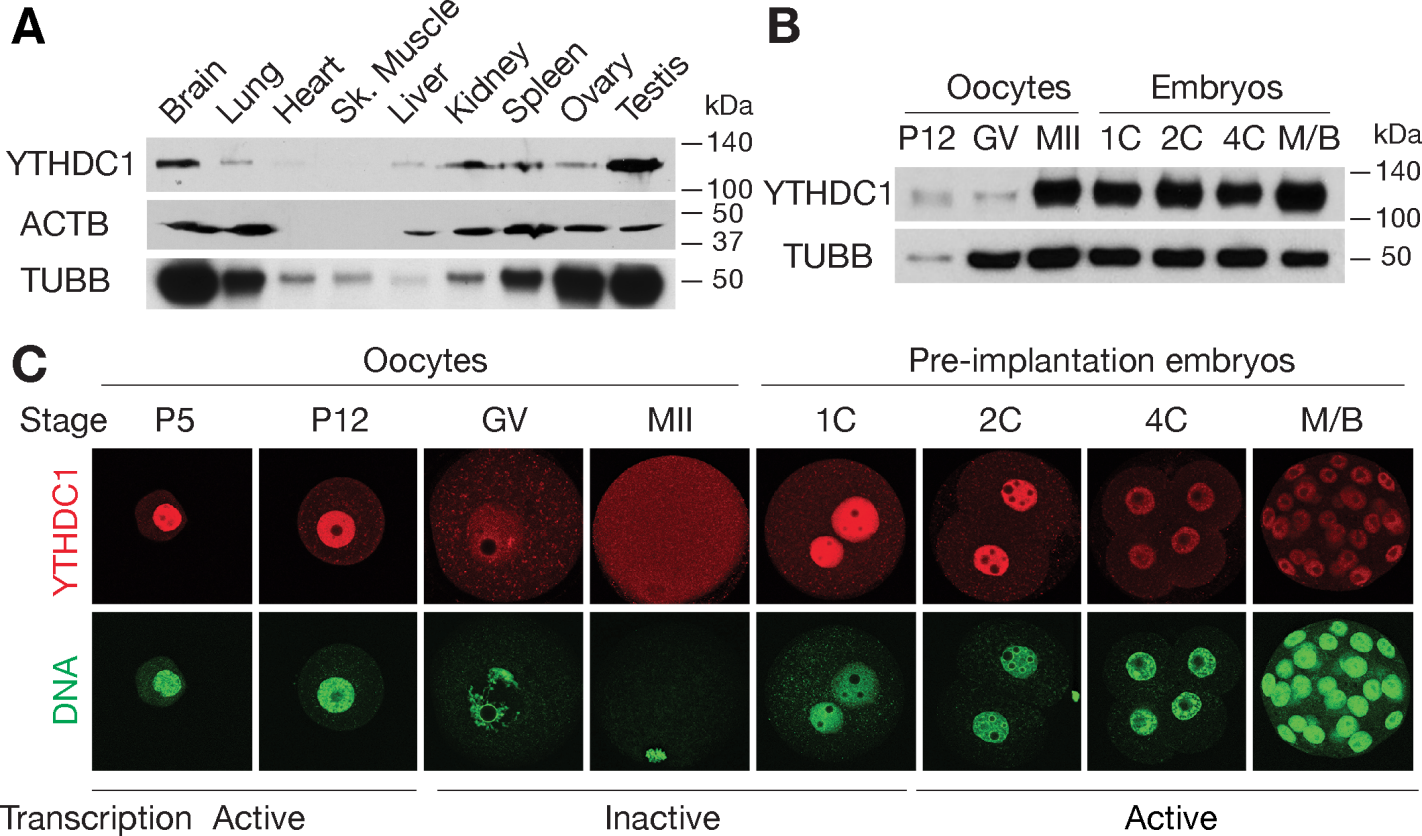
Expression and subcellular localization of YTHDC1 in oocytes and pre-implantation embryos. (A) YTHDC1 expression in adult mouse tissues. ACTB and TUBB (β-tubulin) served as loading controls. Heart and skeletal muscle contain little ACTB. (B) Western blot analysis of YTHDC1 in oocytes and pre-implantation embryos. TUBB served as a loading control. Note the lower levels of YTHDC1 in GV-stage oocytes, when normalized to TUBB. (C) Localization of YTHDC1 in oocytes and pre-implantation embryos. DNA was stained with Sytox green. Abbreviations: P5, P12: postnatal days 5, 12; GV, germinal vesicle stage; MII, metaphase II; 1C, 2C, 4C: 1-cell, 2-cell, 4-cell embryos; M/B, morula/blastocyst.

We examined expression of YTHDC1 in adult mouse tissues using polyclonal antibodies raised against an N-terminal region of mouse YTHDC1 (S2A Fig). Western blot analysis showed that YTHDC1 was expressed in multiple adult mouse tissues including brain, testis, and ovary, with an apparent molecular weight of ~120 kDa (Fig 1A). High levels of YTHDC1 were present in postnatal oocytes, MII eggs, and pre-implantation embryos, and low levels in germinal vesicle (GV) stage oocytes (Fig 1B). The increase in YTHDC1 protein abundance between the GV oocyte stage and MII egg suggests that YTHDC1 is encoded by a dormant maternal mRNA that is recruited during oocyte maturation. Immunostaining showed that YTHDC1 localized to the nucleus in postnatal oocytes and pre-implantation embryos, with the increase in staining between the GV oocyte and MII egg being consistent with the immunoblotting results (Fig 1C). The diffuse cytoplasmic signal of YTHDC1 and its increased abundance in MII oocytes suggest that *Ythdc1* is under translational control, possibly in preparation for zygotic activation at the two-cell stage. The nuclear localization of YTHDC1 is consistent with a previous finding that it is associated with chromatin [57]. Notably, in postnatal day (PND) 5 and 12 oocytes, transcription is active and YTHDC1 is nuclear. In adult testis (S1 Fig), YTHDC1 is nuclear in spermatogonia, spermatocytes, and round spermatids, which are transcriptionally active. However, YTHDC1 is absent in elongating and elongated spermatids, which are transcriptionally inactive due to nuclear condensation (S1 Fig). Therefore, the nuclear localization of YTHDC1 in cells with active transcription suggests that it is involved in co-transcriptional and/or post-transcriptional regulations.

### YTHDC1 is required for embryo viability

To determine the physiological function of *Ythdc1*, we generated a *Ythdc1* floxed (conditional) allele, referred to as *Ythdc1*^fl^, by gene targeting in embryonic stem (ES) cells (S2B Fig). *Ythdc1*^fl/fl^ mice were healthy and fully fertile. We next crossed *Ythdc1*^fl/fl^ mice to *Actb*-Cre mice, which express Cre ubiquitously, to obtain mice with a *Ythdc1* null allele (*Ythdc1*^+/-^) [60]. Cre-mediated excision of the floxed exons removes the YTH domain and causes a frameshift in the resulting *Ythdc1* mutant transcript (S2B Fig). Intercrosses of *Ythdc1*^+/-^ mice did not produce any *Ythdc1*^-/-^ pups, suggesting that *Ythdc1* is essential for embryonic development (S2C Fig). To determine the time of developmental failure, we genotyped fetuses recovered from intercrosses of *Ythdc1*^+/-^ mice at embryonic day 8.5 (E8.5), E9.5, and E11.5. No *Ythdc1*^-/-^ embryos were found at E11.5. Out of 9 embryos at E8.5 and out of 33 embryos at E9.5, only one *Ythdc1*^-/-^ embryo was found at each time point (S2C Fig). Resorbed embryos were found at E8.5 through E11.5 and expected to be homozygous mutants based on their Mendelian distribution (S2C Fig). These results show that YTHDC1 is indispensable for embryo development past early post-implantation stages.

### YTHDC1 is essential for spermatogonium survival and male fertility

**Fig 2:**
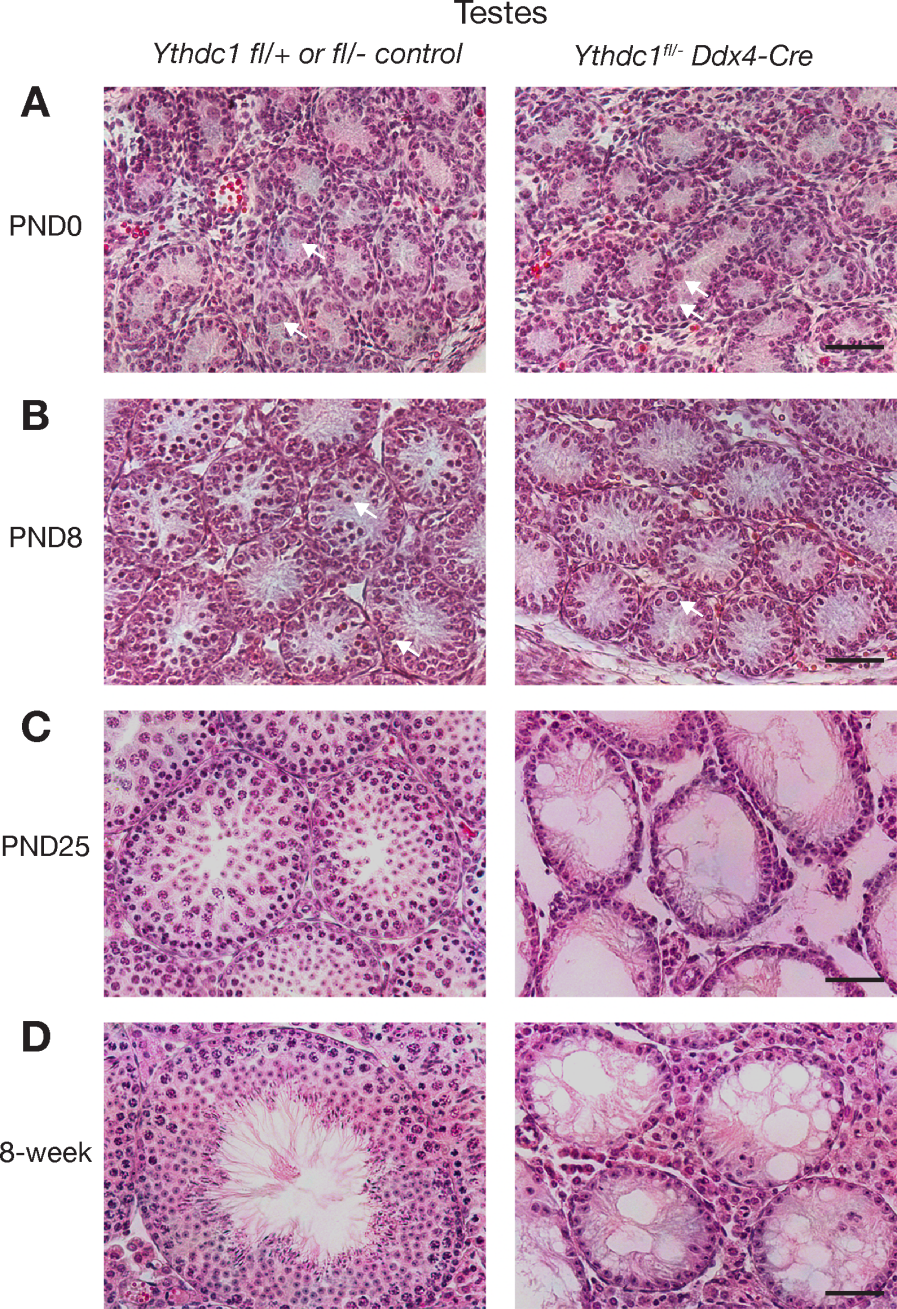
Postnatal loss of male germ cells in *Ythdc1*^fl/-^ *Ddx4*-Cre males. Histological analysis of testes from *Ythdc1* wild-type or heterozygous (left) and *Ythdc1*^fl/-^ *Ddx4*-Cre (right) males at birth (postnatal day 0) (A), PND8 (B), PND25 (C), and 8 weeks (D). Arrows in panels A and B indicate prospermatogonia and spermatogonia respectively. Scale bars, 50 μm.

**Fig 3:**
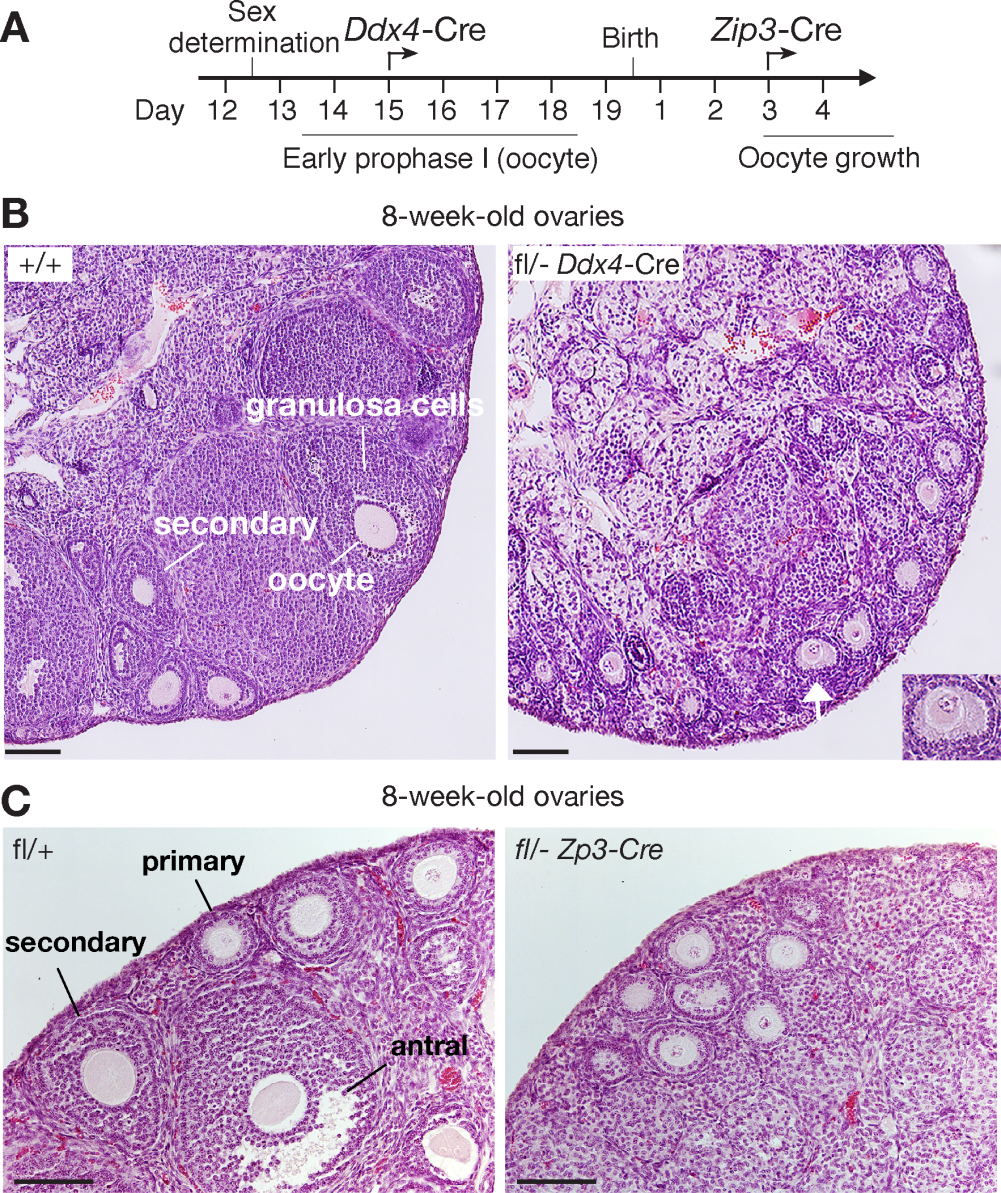
YTHDC1 is required for oocyte growth. (A) Timeline of disruption of *Ythdc1* in oocytes using *Ddx4*-Cre or *Zp3*-Cre. (B) Histological analysis of ovaries from 8-week-old wild-type and *Ythdc1*^fl/-^ *Ddx4*-Cre mice. Inset, enlarged view of the primary follicle marked by white arrow. Scale bars, 100 μm. (C) Histological analysis of ovaries from 8-week-old wild-type and *Ythdc1*^fl/-^ *Zp3*-Cre mice. Scale bars, 100 μm.

To bypass the embryonic lethality resulting from *Ythdc1* deficiency, we used *Ddx4*-Cre to inactivate *Ythdc1* specifically in the germline to generate *Ythdc1*^fl/-^ *Ddx4*-Cre (referred to as *Ythdc1*^cKO^) mice (Figs 2 and 3A). All subsequent studies were conducted with *Ythdc1*^fl/-^ *Ddx4*-Cre (cKO) mice unless noted otherwise. *Ddx4*-Cre expression begins at ~E15 in both male and female germ cells but differs in the developmental stage of onset due to the sexual dimorphism in the timing of meiotic entry [61]. In males, *Ddx4-Cre* is expressed in mitotic germ cells including spermatogonia prior to meiosis, whereas in oocytes, *Ddx4*-Cre expression occurs only after meiotic entry (Fig 3A). *Ythdc1*^cKO^ mice were viable and grossly normal. Seminiferous tubules from newborn (PND0) *Ythdc1*^cKO^ males contained prospermatogonia (Fig 2A), which lacked YTHDC1 as determined by immunostaining (S3 Fig). Tubules from PND8 *Ythdc1*^cKO^ males contained substantially fewer spermatogonia than those from control *Ythdc1*^fl/+^ or *Ythdc1*^fl/-^ males (Fig 2B). However, testes from PND25 and adult *Ythdc1*^cKO^ males lacked any germ cells including mitotic spermatogonia and exhibited a Sertoli-cell-only phenotype (Fig 2C and 2D), demonstrating that *Ythdc1* is required for development of spermatogonia and male fertility.

### Inactivation of YTHDC1 causes oocyte maturation arrest and female sterility

In contrast to the absence of germ cells in adult *Ythdc1* cKO testis, oocytes were present in ovaries from 8-week-old *Ythdc1*^fl/-^ *Ddx4*-Cre (cKO) females (Fig 3). Wild-type adult ovaries contained follicles at different developmental stages, including primary, secondary, and antral follicles (Fig 3B and 3C). However, *Ythdc1*^fl/-^ *Ddx4*-Cre ovaries lacked secondary or antral follicles, indicating that oocyte development was blocked at the primary follicle stage, which is characterized by one layer of granulosa cells surrounding the oocyte (Fig 3B). Histological analysis of ovaries from older *Ythdc1*^fl/-^ *Ddx4*-Cre females (6-month and beyond) showed a complete loss of oocytes. Western blot analysis confirmed that YTHDC1 protein was absent in oocytes collected from *Ythdc1*^fl/-^ *Ddx4*-Cre ovaries (S4A Fig). As expected, a nuclear immunofluorescent signal of YTHDC1 was not detected in *Ythdc1* mutant oocytes (S4B Fig). These results confirm the specificity of our YTHDC1 antibody and the complete depletion of YTHDC1 in *Ythdc1*^fl/-^ *Ddx4*-Cre oocytes.

Because expression of *Ddx4*-Cre begins at the pachytene stage of meiotic prophase I during fetal development, it is not clear whether the observed defects in *Ythdc1*^fl/-^ *Ddx4*-Cre postnatal ovaries were due to the requirement of YTHDC1 at embryonic or postnatal stages. To investigate whether postnatally expressed YTHDC1 is required for oocyte development, we used *Zp3*-Cre to inactivate *Ythdc1* in oocytes postnatally (Fig 3A). *Zp3*-Cre is expressed in developing oocytes around postnatal day 3 (Fig 3A) [62]. We found that *Ythdc1*^fl/-^ *Zp3*-Cre ovaries exhibited similar defects in folliculogenesis as observed in *Ythdc1*^fl/-^ *Ddx4*-Cre ovaries–blockade at the primary follicle stage (Fig 3C). We next performed mating tests of three *Ythdc1*^fl/-^ *Zp3*-Cre females and three wild-type littermate control females. At the age of 8 weeks, each female was housed with one wild-type male for two months. The three control females produced two litters each (6.8 ± 1.4 pups/litter), whereas none of the three *Ythdc1*^fl/-^ *Zp3*-Cre females produced any offspring. Taken together, these genetic studies demonstrate that YTHDC1 plays an essential role in postnatal oocyte development.

### *Ythdc1*-deficient oocytes contain large cytoplasmic RNA granules

**Fig 4:**
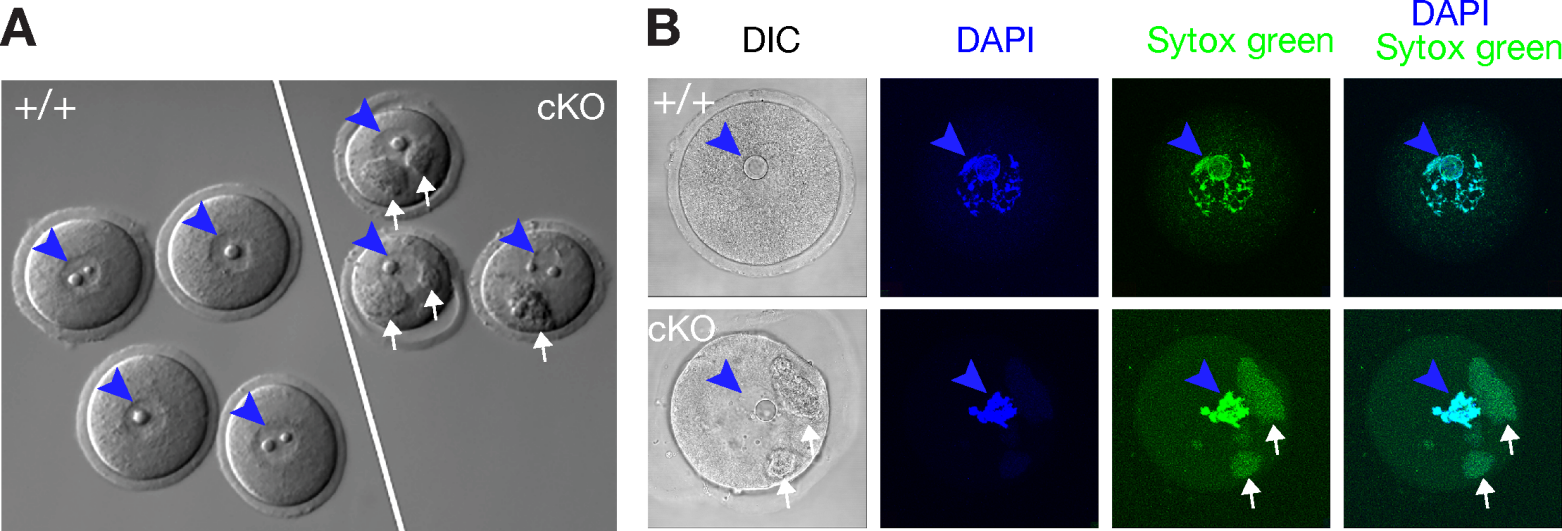
RNA-containing cytoplasmic granules in *Ythdc1*-deficient oocytes. (A) Presence of large cytoplasmic granules in oocytes from 11-week-old *Ythdc1*^fl/-^ *Ddx4*-Cre (cKO) females. (B) Cytoplasmic granules in oocytes from 11-week-old *Ythdc1*^fl/-^ *Ddx4*-Cre females contain RNA. Nuclei/nuclear DNA and cytoplasmic RNA granules are marked by arrowheads (blue) and arrows (white), respectively. DAPI stains DNA only. Sytox green stains both DNA and RNA.

We were able to retrieve oocytes from ovaries of *Ythdc1*^fl/-^ *Ddx4-*Cre females at the ages of 3–6 weeks by poking. However, the number of oocytes retrieved from *Ythdc1*^fl/-^ *Ddx4-*Cre females was only 10% (n = 3) that of wild-type littermates. In addition, the GV oocytes from *Ythdc1*^fl/-^ *Ddx4-*Cre females were not able to resume meiosis in vitro. In contrast to the smooth appearance of wild-type germinal vesicle (GV) stage oocytes, *Ythdc1*-deficient oocytes contained one or two prominent granules in the cytoplasm (Fig 4A). Such granules were not observed in wild-type oocytes. These granules stained positive with Sytox green, which recognizes both DNA and RNA, suggesting a nucleic acid content (Fig 4B). When double stained with both DAPI and Sytox green, the nuclei of *Ythdc1*-deficient oocytes were positive for both stains, whereas the granules only retained the Sytox green stain, indicating that the granules contained RNA but not DNA (Fig 4B). To our knowledge, such large RNA granules have not been observed before. The appearance of large cytoplasmic RNA granules indicates severe defects in RNA metabolism in oocytes in the absence of YTHDC1. It is possible that, like P granules, incorrectly processed RNAs are sequestered in these novel RNA granules in oocytes in the absence of YTHDC1. Knockdown of YTHDC1 in HeLa cells causes acute nuclear accumulation of mRNAs within hours [59]. We did not observe nuclear accumulation of RNAs in postnatal *Ythdc1*-deficient oocytes, possibly because inactivation of *Ythdc1* begins at E15 (Fig 3A), weeks prior to our analysis.

To investigate the consequences of *Ythdc1* deficiency on the oocyte transcriptome, we performed RNA-seq analysis of oocytes collected from 6-week-old wild-type and *Ythdc1*^fl/-^ *Ddx4-*Cre females (S1 Table). With a FDR cutoff of 0.01, a total of 4933 transcripts showed differential expression: 2656 transcripts were up-regulated and 2277 transcripts down-regulated in *Ythdc1*-deficient oocytes compared with control oocytes, indicating that the transcriptome in *Ythdc1*-deficeint oocytes was dramatically altered (S5A Fig and S2 Table). Validation of 10 randomly selected differentially abundant transcripts by real-time PCR confirmed the RNA-seq findings (S5B Fig). Gene Ontology (GO) analysis identified a number of significantly altered biological processes for both up-regulated and down-regulated transcripts, with regulation of transcription as the most significantly affected process (S5C Fig).

### Widespread splicing defects in *Ythdc1*-deficient oocytes

**Fig 5:**
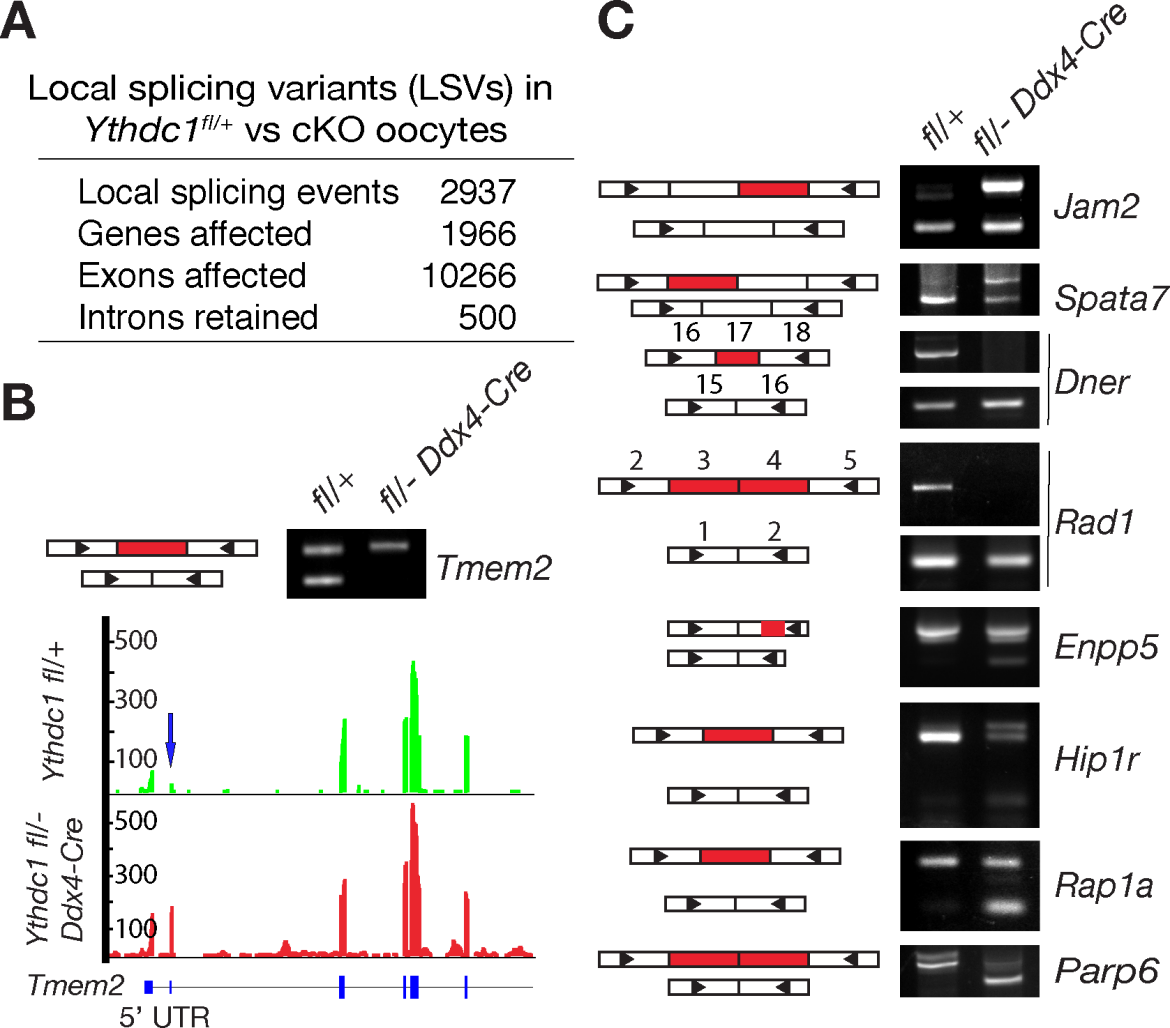
Changes in the splicing landscape in *Ythdc1*-deficient oocytes. Oocytes were collected from 6-week-old *Ythdc1*^fl/+^ and *Ythdc1*^fl/-^ *Ddx4*-Cre females. (A) Summary of local splicing variants (LSVs) identified by MAJIQ. Significant LSVs: ΔPSI (difference in percentage spliced in) > 0.2 and q < 0.05. (B) PCR validation and gene track view of one exon-skipping LSV in *Tmem2*. (C) PCR validation of LSVs affecting internal exons. Exons are represented as rectangles but not in scale. Skipped or retained exons are shown in red. Triangles denote the positions of PCR primers. Each PCR assays was performed three times using different samples.

Because YTHDC1 affects alternative splicing in cultured somatic cells [27], we next analyzed the oocyte RNA-seq data to systematically identify local splicing variants (LSVs) between wild-type and *Ythdc1*-deficient oocytes using the MAJIQ package [63]. We identified a total of 2937 significant LSVs (q < 0.05) with ΔPSI (difference in percent spliced in) > 0.2 (Fig 5A and S3 Table). These LSVs affected 1966 genes, involved 10,266 exons, and included differential retention of 500 introns. Of the 1966 genes with LSVs, 34% (659 genes) were differentially expressed between wild-type and *Ythdc1*-deficient oocytes (up-regulated, 245 genes; down-regulated, 414 genes). According to GO analysis, these changes affect genes involved in multiple fundamental biological processes, including chromatin modification, regulation of transcription, mRNA processing, and regulation of translation (S6 Fig).

We designed RT-PCR assays to validate different types of MAJIQ-identified splicing events: exon inclusion/skipping, intron retention, and splicing in 3’ UTRs. Using GV-stage oocytes from wild-type and *Ythdc1*^fl/-^ *Ddx4*-Cre mice, RT-PCR analysis confirmed 90% (9 of 10 tested) of LSVs involving internal exons (Fig 5B and 5C). For example, as illustrated in the gene track view, the second exon (part of the 5’UTR) of *Tmem2* was partially skipped in wild-type but not in *Ythdc1*-deficient oocytes (Fig 5B). Two LSVs affecting *Jam2* and *Spata7*, respectively, involved nearly complete exon skipping in wild-type oocytes but partial exon skipping in *Ythdc1*-deficient oocytes. Exon skipping in two genes (*Dner* and *Rad1*) was complete in *Ythdc1*-deficient oocytes but partial in wild-type. Four LSVs in *Enpp5*, *Hip1r*, *Rap1a*, and *Parp6* resulted in partial exon skipping in *Ythdc1*-deficient oocytes whereas no skipping occurred in wild-type. There was no apparent preference for the directionality of exon skipping in regard to genotype. In total, our validation results suggest that most LSVs predicted by MAJIQ are true splicing events.

**Fig 6:**
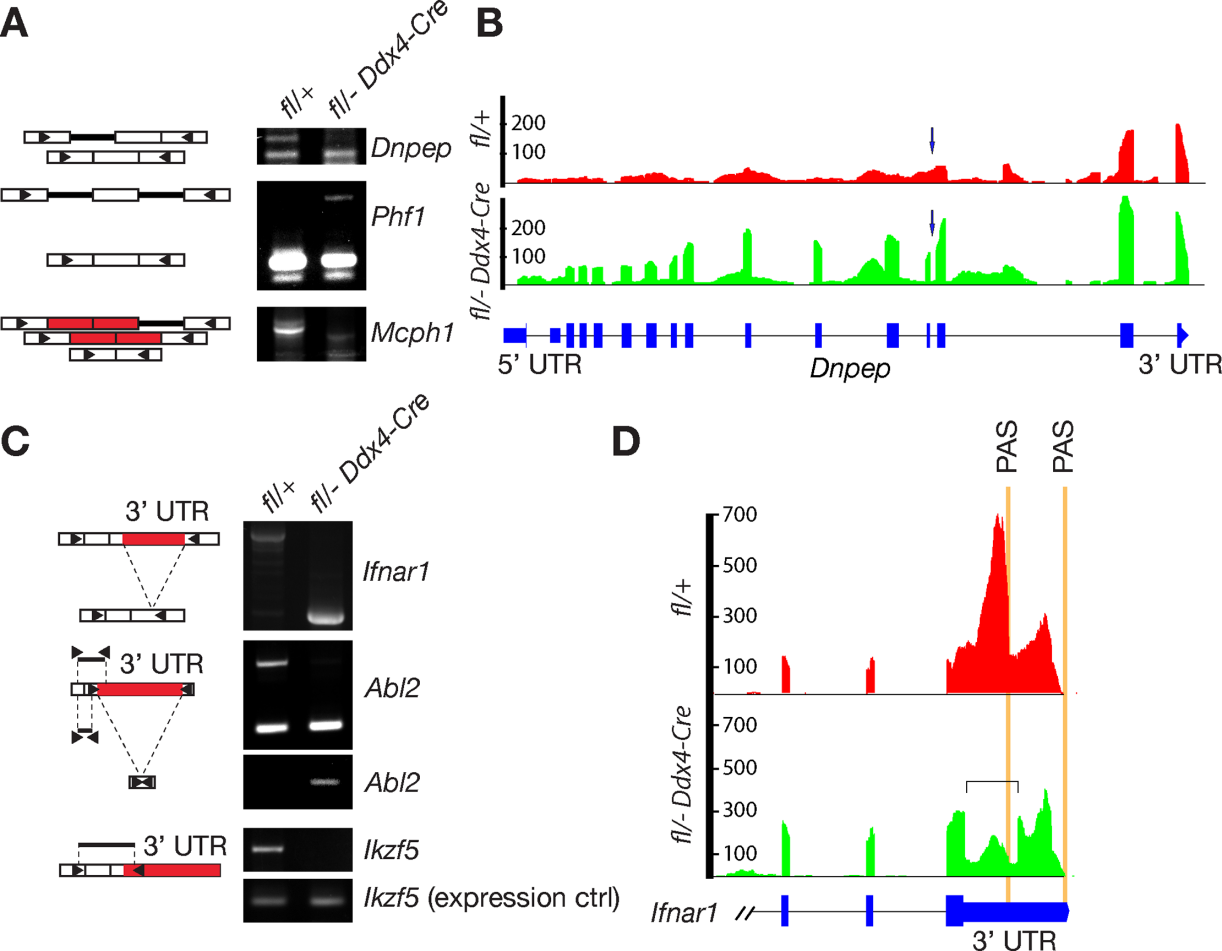
Local splicing variants involve intron retention and 3’ UTR. Oocytes were collected from 6-week-old *Ythdc1*^fl/+^ and *Ythdc1*^fl/-^ *Ddx4*-Cre females. (A) PCR validation of intron-retaining LSVs. Introns and exons are represented as thick lines and rectangles, respectively. (B) Gene track view of a retained intron in the *Dnpep* Gene. Arrow indicates the affected intron. (C) PCR validation of LSVs in 3’ UTRs. The affected portion of the respective 3’ UTR is shown in red. Triangles denote the positions of PCR primers (A and C). (D) Gene track view of splicing variant in the *Ifnar1* 3’ UTR. The square bracket demarcates the spliced region of 3’ UTR in *Ythdc1*-deficient oocytes. Polyadenylation sites (PAS) are marked by vertical orange lines.

LSVs involving introns or 3’ UTRs were more complex. We tested nine intron-retention LSVs predicted by MAJIQ and confirmed six of these (67%) by RT-PCR (Fig 6A and 6B). Among three confirmed LSVs, two transcripts (*Dnpep* and *Mcph1*) showed intron retention preferentially in wild-type oocytes, whereas one mRNA (*Phf1*) retained introns preferentially in *Ythdc1*-deficient oocytes. We examined 13 MAJIQ-predicted LSVs involving splicing within 3’ UTRs and validated three of these (23%) by RT-PCR (Fig 6C and 6D): *Ifnar1*, *Abl2*, and *Ikzf5*. Coincidentally, all of these transcripts were associated with longer 3’ UTRs in wild-type oocytes. Although MAJIQ was not designed for the analysis of changes in introns and 3’ UTR length as pointed out by the MAJIQ authors, we were able to validate most of the MAJIQ-predicted LSVs involving introns and some of the 3’ UTR splicing events.

We further examined the 9 LSVs involving exon inclusion/skipping using GV-stage oocytes from 6-week-old *Ythdc1*^fl/-^ *Zp3*-Cre and wild-type females. All 9 LSVs validated in *Ythdc1*^fl/-^ *Ddx4*-Cre oocytes (Fig 5) were also confirmed in *Ythdc1*^fl/-^ *Zp3*-Cre oocytes (S7 Fig). Collectively, our results show that inactivation of YTHDC1 in oocytes causes severe defects in mRNA splicing.

### Extensive alternative polyadenylation in *Ythdc1*-deficient oocytes

**Fig 7:**
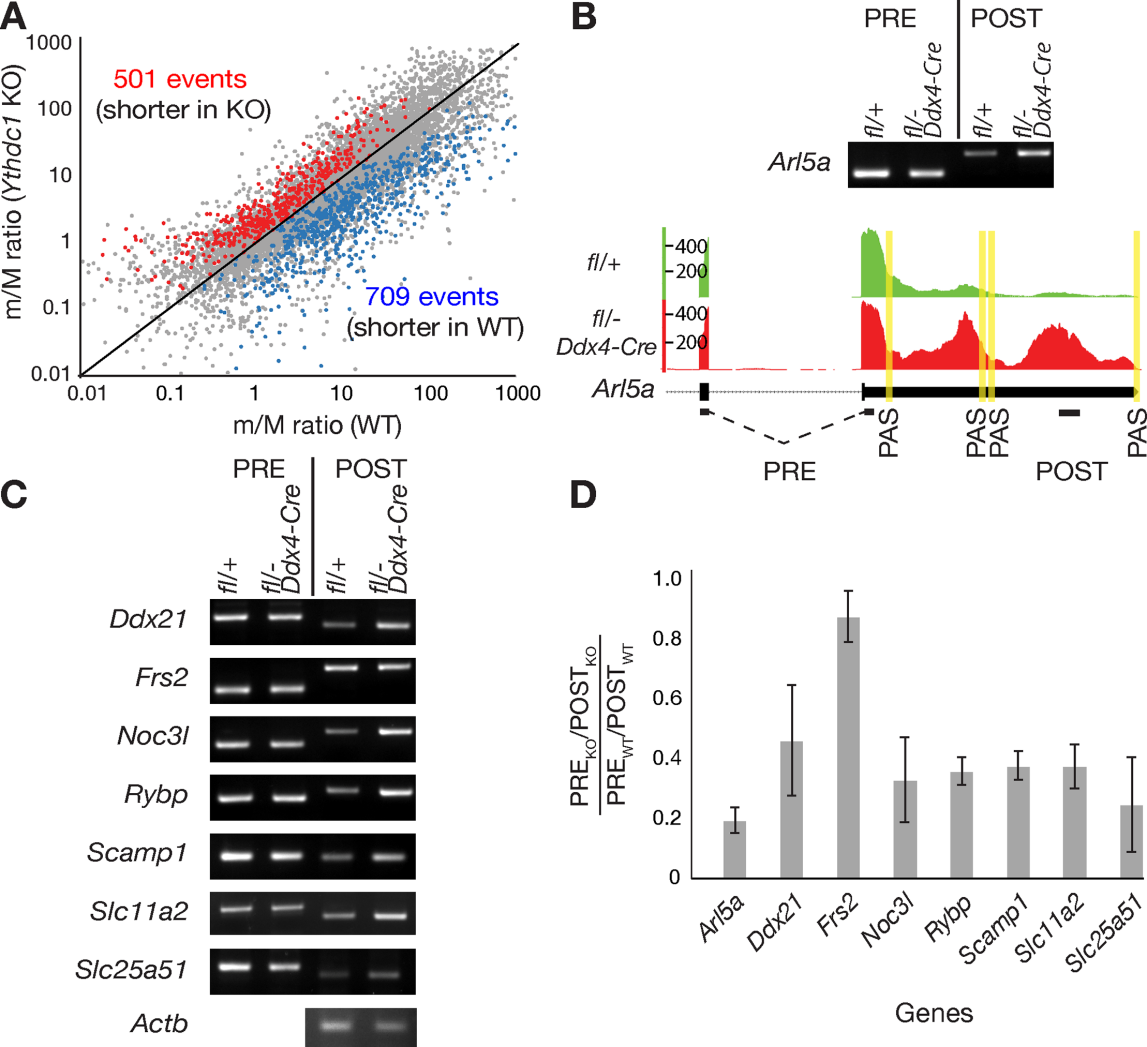
Alternative polyadenylation in *Ythdc1*-deficient oocytes. Oocytes were collected from 6-week-old *Ythdc1*^fl/+^ and *Ythdc1*^fl/-^ *Ddx4*-Cre females. (A) Pairwise comparison of PAS usage in wild-type and *Ythdc1*-deficient oocytes. PAS pairs with p<0.05 are shown in red or blue. (B) Gene track view and RT-PCR validation of alternative polyadenylation in *Arl5a*. Polyadenylation sites (PAS) are marked by vertical yellow lines. Exons and introns are marked by black bars and dotted lines, respectively, and the location of PRE and POST PCR fragments is shown. (C) RT-PCR validation results of alternative polyadenylation in 7 genes. *Actb* served as a loading control. (D) Quantification of RT-PCR products of 8 genes shown in panels A and B. A ratio [(PRE_KO_/POST_KO_)/(PRE_WT_/POST_WT_)] less than 1 indicates a higher level of the long isoform (with a longer 3’ UTR) in *Ythdc1*-deficient oocytes. A ratio of 1 for *Frs2* indicates no preference between wild-type and mutant. Y-axis: mean ± SD. The experiments were performed in triplicates.

The majority of m^6^A sites are present in the 3’ most exons, raising the possibility that m^6^A may play a role in regulating 3’ UTR length [5, 9]. Many genes produce transcripts with 3’ UTRs of different lengths due to usage of alternative polyadenylation (APA) sites and 3’ UTRs contain sites for microRNAs and RNA-binding proteins. Thus, the 3’ UTR of a particular mRNA regulates its translation and subcellular localization [64, 65]. For instance, transcripts in brain exhibit extensive lengthening of 3’ UTRs due to APA [66]. We systematically analyzed the 3’ UTR length of wild-type versus *Ythdc1*-deficient oocytes using the ROAR algorithm [67]. The ROAR program identifies alternative polyadenylation using standard RNA-seq data by measuring the reads upstream (pre) and downstream (post) of the annotated polyadenylation site (PAS) (Figs 7A and 6B). ROAR analysis of our oocyte RNA-seq data revealed 1210 alternative polyadenylation (APA) events in 864 genes between wild-type and *Ythdc1* mutant oocytes (cutoff, p value < 0.05; Fig 7A and S4 Table). Some genes had more than one differential APA event. Overall, 709 APA events (ROAR < 1) resulted in higher levels of the longer isoform (longer 3’ UTR) in *Ythdc1*-deficient oocytes, whereas 501 APA events (ROAR > 1) was associated with higher levels of the shorter isoform in the mutant (Fig 7A).

We chose 8 transcripts with predicted longer 3’ UTRs in the *Ythdc1* mutant for RT-PCR validation (Fig 7B). These 8 transcripts were not differentially expressed between wild-type and mutant oocytes. Our validation strategy involved one PCR assay (termed PRE) that amplified both short and long transcripts and a second PCR assay (termed POST) that was specific for the long isoform. The POST RT-PCR assay for *Arl5a* produced a stronger signal from *Ythdc1*-deficient versus wild-type oocytes, indicating that the former contained a higher level of the long isoform. Of the eight transcripts tested, seven (88%) (*Arl5a*, *Ddx21*, *Noc3l*, *Rybp*, *Scamp1*, *Slc11a2*, and *Slc25a51*) preferentially produced the longer isoform in *Ythdc1*-deficient oocytes due to APA, whereas, one transcript (*Frs2*) did not exhibit detectable differences in isoform prevalence using this assay (Figs 7C and 6D). These results demonstrate extensive alternative polyadenylation in *Ythdc1*-deficient oocytes. Previous findings in brain tissue [9] have shown that five of seven gene transcripts with APA defects contained known m^6^A sites in the last exons: *Arl5a*, *Ddx21*, *Noc3l*, *Slc11a2*, and *Slc25a51*, implicating m^6^A in regulation of alternative polyadenylation.

### m^6^A-dependent rescue of alternative splicing defects in *Ythdc1*-deficient oocytes

**Fig 8:**
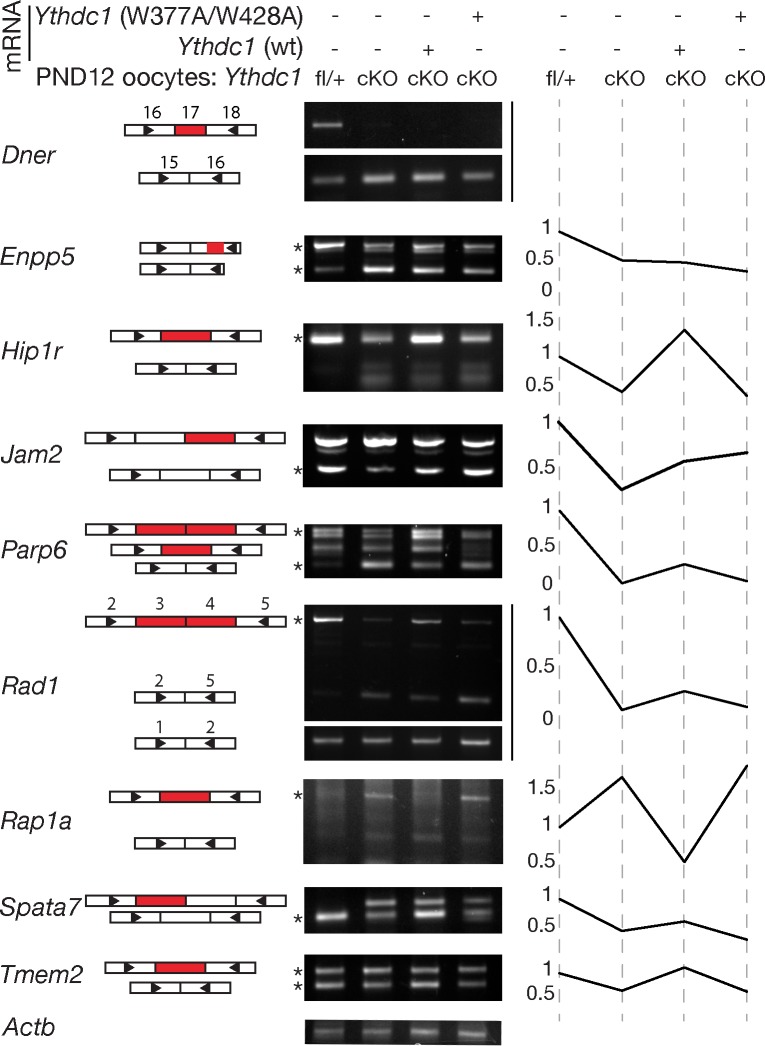
m^6^A-dependent rescue of alternative splicing defects in *Ythdc1*-deficient oocytes. Postnatal day 12 *Ythdc1*^fl/-^ *Ddx4*-Cre (cKO) oocytes were injected with mRNAs encoding wild-type or m^6^A-binding-deficient mutant (W377A W428A) YTHDC1 as marked on top of the gel panel, followed by RT-PCR analysis of LSVs. Left panel, schematic illustration of alternative splicing events for each transcript that correspond to the PCR products shown in the center panel. Each rectangle represents one exon, and exons subject to alternative splicing are marked red. Right panel, plot depicting quantification of ratios of band intensity or a single band intensity, with the value for wild-type oocyte (lane 1) set at 1. Asterisks indicate bands used for quantification. *Enpp5* and *Parp6*: ratio of the upper band / the lower band; *Tmem2*: ratio of the lower band / the upper band. *Actb* serves as a loading control.

To examine the effect of m^6^A on splicing in oocytes, we collected oocytes from wild-type and *Ythdc1*^fl/-^ *Ddx4*-Cre ovaries at postnatal day 12 (PND12), when oocytes are still transcriptionally active. We evaluated alternative splicing of the nine transcripts for which we had identified splicing defects in GV stage oocytes from 6-week-old *Ythdc1*^fl/-^ *Ddx4*-Cre mice (Fig 5) and found that all nine transcripts showed similar splicing defects in PND12 *Ythdc1*-deficient oocytes (Fig 8, first two lanes of center panel). However, there were notable differences for two transcripts: *Rad1* and *Tmem2*. The *Rad1* two-exon-skipping isoform was detected in PND12 mutant oocytes (Fig 8) but not in 6-week-old mutant oocytes (Fig 5). Similarly, the *Tmem2* spliced short isoform was present in PND12 mutant oocytes but absent in 6-week-old oocytes. These data suggest that the short isoforms of *Rad1* and *Tmem2* were degraded during the long period between cessation of transcription at PND20 and the time point of analysis at 6 weeks-of-age.

To investigate if alternative splicing defects in *Ythdc1*-deficient oocytes could be rescued by supplying YTHDC1, we used transcriptionally active PND12 oocytes. We injected PND12 *Ythdc1*-deficient oocytes with *in vitro* transcribed wild-type or mutant *Ythdc1* mRNA, followed by overnight culture. The *Ythdc1* mutant mRNA contains two missense mutations (W377A, W428A) that completely abolish the m^6^A binding activity of YTHDC1 [24]. We quantified the FLAG-YTHDC1 protein levels in the nucleus of injected *Ythdc1*-deficient oocytes by immunofluorescence and confocal microscopy and found no difference in YTHDC1 protein levels between oocytes (4 oocytes each) injected with wild-type and mutant *Ythdc1* mRNAs. We found that exon skipping and exon inclusion defects in six transcripts were rescued in *Ythdc1*-deficient oocytes injected with wild-type but not mutant *Ythdc1* mRNA, whereas no difference was observed for the remaining three genes (*Dner*, *Enpp5* and *Jam2*) (Fig 8). These rescue experiments suggest that the majority of alternative splicing defects in *Ythdc1*-deficient oocytes is m^6^A-dependent. However, we cannot rule out the possibility that the failure of mutant YTHDC1 ((W377A, W428A) to rescue might be caused by reduced RNA-binding activity or instability, independent of m^6^A.

### YTHDC1 interacts with pre-mRNA 3’end processing factors

**Fig 9:**
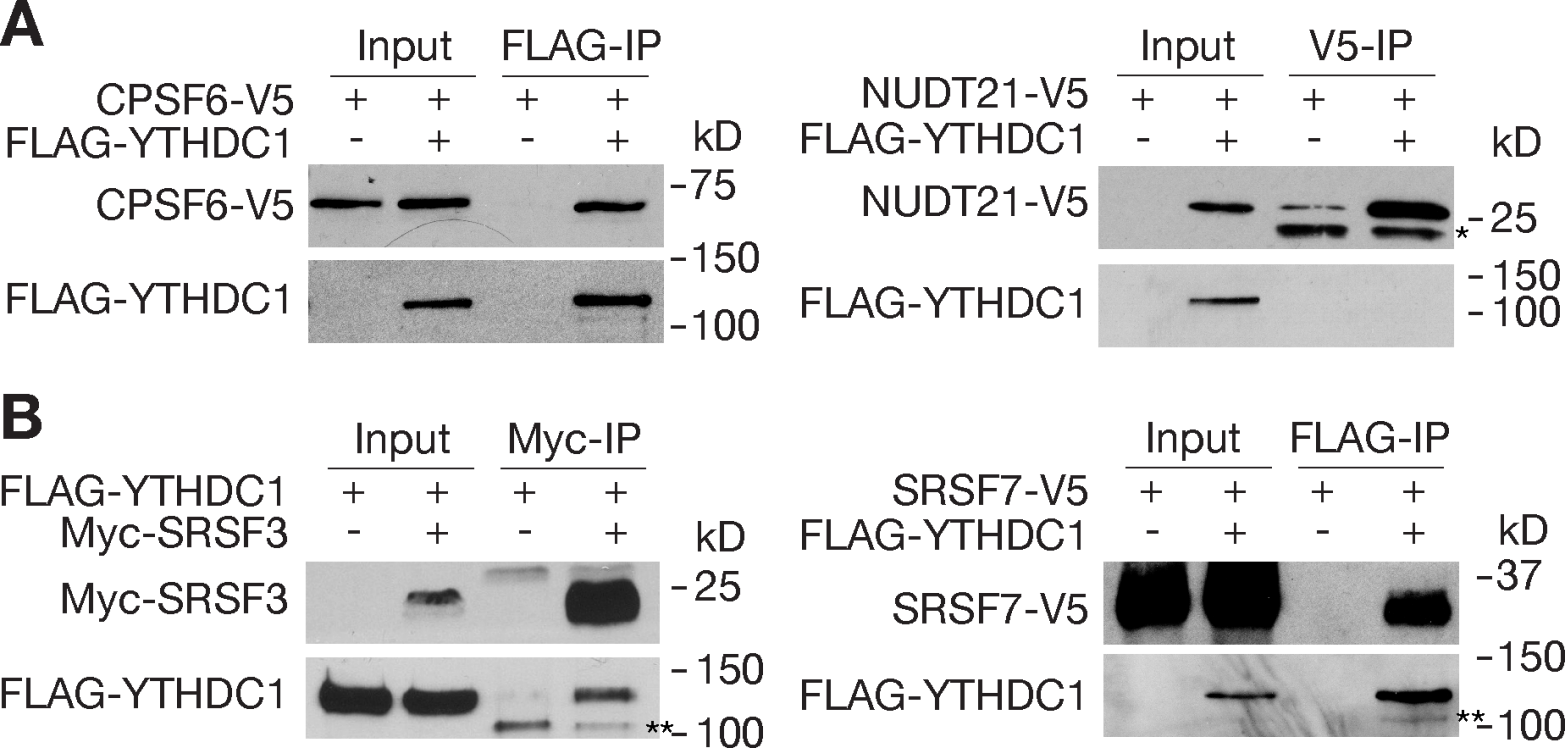
Association of YTHDC1 with pre-mRNA 3’end processing factors. Recombinant proteins were expressed in HEK 293T cells. Co-immunoprecipitation was carried out in the presence of RNase. (A) Co-IP analysis of YTHDC1 with CPSF6 and NUDT21. * indicates antibody light chain. (B) Co-IP analysis of YTHDC1 with SRSF3 and SRSF7. ** indicates a non-specific band.

To elucidate the mechanism by which YTHDC1 affects alternative polyadenylation, we investigated potential interactions of YTHDC1 with pre-mRNA 3’end cleavage and polyadenylation factors by co-immunoprecipitation (Fig 9). Cleavage factor Im (CFIm) and cleavage stimulating factor (CSTF) are two multi-protein complexes that bind to upstream sequence elements (USE) and downstream sequence elements (DSE) around the PAS, respectively [68, 69]. We found that YTHDC1 was associated with CPSF6, one of the four subunits of the CFIm complex (Fig 9A). This result is consistent with previous reports identifying CPSF6 among proteins co-immunoprecipitated with human YTHDC1 in 293T cells [46]. However, YTHDC1 did not interact with NUDT21, another subunit of the CFIm complex (Fig 9A). In addition, YTHDC1 was not associated with cleavage stimulating factors CSTF1 or CSTF2 by co-transfection and co-IP assays. Interestingly, knockdown of *Cpsf6* induces widespread use of proximal PAS, resulting in 3’ UTR shortening [70, 71]. Moreover, a mutation in the Medaka *Cpsf6* gene causes 3’ UTR shortening in developing embryos and a defect in primordial germ cell migration [72].

YTHDC1 interacts with the SR splicing factors SRSF3 and SRSF7 (Fig 9B). SRSF3 and SRSF7 couple RNA processing with mRNA export through association with the nuclear mRNA export factor NXF1 [73]. SRSF3 and SRF7 bind to the last exons and regulate polyadenylation in an opposing manner. Knockdown of SRSF3 leads to 3’ UTR shortening, whereas depletion of SRSF7 results in 3’ UTR lengthening [73]. In conclusion, these results support a model in which YTHDC1 regulates alternative polyadenylation through interaction with the 3’end processing machinery.

## Discussion

Here, we report that the nuclear m^6^A reader YTHDC1 is essential for mouse embryogenesis and germline development, and describe a critical role of YTHDC1 in orchestrating m^6^A-dependent processing of pre-mRNA transcripts in oocytes. Our studies implicate YTHDC1 in the choice of polyadenylation sites, which determines the length of 3’ UTRs. The 3’ UTR contains target sites for microRNAs and many RNA-binding proteins. Therefore, lengthening or shortening of 3’ UTR would predictably have profound effects on translation efficiency, transcript stability, and subcellular transcript localization [64, 65, 68, 69]. Precise translational control of maternal transcripts is especially critical during oocyte maturation, due to lack of transcription during this prolonged stage. We find that loss of YTHDC1 in oocytes results in alternative polyadenylation and thus altered 3’ UTR length in more than 800 genes. To date, YTHDC1 is the only m^6^A reader that has been demonstrated to regulate 3’ UTR length.

Triple knockdown of three m^6^A writer components (METTL3, METTL14, and WTAP) in human A549 cells changes the usage of proximal versus distal polyadenylation sites with some switching to proximal sites and others switching to distal sites, demonstrating a critical role for m^6^A in regulation of 3’ UTR length [9]. In addition, ALKBH5, an m^6^A demethylase, regulates 3’UTR length in male germ cells [74]. How the m^6^A signal is relayed to the 3’ end processing machinery is unknown. A number of multi-protein complexes participate in pre-mRNA 3’ end cleavage and polyadenylation, including cleavage factor Im (CFIm), cleavage and polyadenylation specificity factor (CPSF), cleavage stimulating factor (CSTF), and poly(A)-binding proteins [68, 69]. We find that YTHDC1 forms complexes with components of the 3’ end processing machinery: CPSF6 (a CFIm component), SRSF3, and SRSF7 (Fig 9). These factors bind to the 3’ UTR around the PAS. Specifically, the CFIm binds to the UGUA motif upstream of the PAS. Knockdown of each of these factors in cell culture causes a shift in PAS usage, resulting in APA. Knockdown of CPSF6 favors usage of proximal PAS [70, 71], whereas knockdown of SRSF7 causes preferential usage of distal PAS [73]. Our data support a model in which YTHDC1 recognizes m^6^A in the last exons of pre-mRNA transcripts and orchestrates the choice of polyadenylation sites through interactions with 3’end processing factors. YTHDC1 may recruit these factors to the 3’ UTRs or sequester them in the nucleoplasm through interactions, resulting in opposing APA patterns. Alternatively, these factors may compete for binding to YTHDC1. In addition, SRSF3 and SRSF7 link alternative polyadenylation with nuclear export through interaction with NXF1 [73]. Therefore, it is conceivable that, through interaction with SRSF3 and SRSF7, YTHDC1 may couple m^6^A in alternatively polyadenylated transcripts with nuclear export. In cultured cells, YTHDC1 facilitates binding of m^6^A-modified nuclear transcripts to SRSF3 and NXF1 and mediates nuclear export [59].

Our study reveals an essential role for YTHDC1 in development of both the embryo and the germline. Proteins (writers, readers, and erasers) involved in establishment, recognition, and erasure of m^6^A sites in mRNAs play important roles in development and fertility in mouse. Loss of the key m^6^A writer enzyme METTL3 causes early post-implantation lethality with defects in lineage priming [49]. m^6^A mainly reduces mRNA stability in embryonic stem cells and pre-implantation embryos and its loss leads to a failure in termination of naïve pluripotency during lineage specification [49]. Conditional inactivation of *Mettl3*/*Mettl14* reveals their essential role in spermatogenesis [50, 51]. *Alkbh5*-deficient mice are viable but exhibit impaired spermatogenesis with increased apoptosis of meiotic spermatocytes [22]. ALKBH5-mediated m^6^A demethylation affects mRNA export. The cytoplasmic m^6^A reader YTHDC2 interacts with the meiosis-specific protein MEIOC [29, 30]. *Ythdc2*-deficient mice are viable but sterile due to a failure in meiotic progression [26, 31–33]. YTHDC2 together with MEIOC promotes translation efficiency of its target transcripts but decreases their mRNA abundance. In addition, YTHDC2 modulates the level of m^6^A-enriched transcripts in germ cells, which is required for progression through meiosis [32]. *Ythdf2* deficiency causes incomplete penetrance of lethality and female-specific infertility [53].

Similar to *Mettl3*, inactivation of *Ythdc1* is embryonic lethal, showing that loss of YTHDC1 is not compensated for by other m^6^A readers. The lack of compensation is not entirely surprising, given that, to date, YTHDC1 is the only known m^6^A reader in the nucleus. By conditional inactivation of *Ythdc1* in the germline, we find that YTHDC1 is essential for fertility in both males and females. Specifically, YTHDC1 is required for development of mitotic spermatogonia in males and oocyte growth in females. Because of loss of spermatogonia in *Ythdc1*^fl/-^ *Ddx4*-Cre males, different Cre drivers will be needed to examine its role in meiotic spermatocytes and post-meiotic round spermatids in future studies. Strikingly, the mouse mutant phenotypes of three m^6^A readers YTHDC1, YTHDC2, and YTHDF2 are different, suggesting non-redundant functions. YTHDC2 is required for meiotic progression in both sexes but is dispensable for viability [26, 31–33]. YTHDF2 is partially necessary for viability and specifically required for female fertility and oocyte competence [53]. Here we find that YTHDC1 is essential for viability and is required for spermatogonial development in males and oocyte growth in females. About 200 transcripts were upregulated in *Ythdf2*-deficient MII oocytes [53]. The overlap between the upregulated transcripts in *Ythdf2*-deficient oocytes and *Ythdc1*-deficient oocytes is significant (1.48-fold enrichment, p = 0.0004), suggesting that YTHDC1 may play a later role in oocyte competence. In *Ythdc1*^fl/-^ *Ddx4*-Cre or *Ythdc1*^fl/-^ *Zp3*-Cre females, germ cells progress through the prophase of meiosis I, however, oocyte development is blocked at the primary follicle stage. This blockade is similar to the oocyte growth arrest in females lacking GDF9, a key TGFβ receptor ligand [75].

Several early studies using cultured cells show that YTHDC1 is involved in alternative splicing of internal exons in a dosage–dependent manner [27, 28, 58]. YTHDC1 localizes to so-called YT bodies in the nucleus that contain active transcription sites. Tyrosine phosphorylation of YTHDC1 regulates its solubility in the nucleus and its effect on alternative splicing. Structural demonstration of YTHDC1 as an m^6^A reader raises a possible connection between m^6^A and alternative splicing [24, 25]. Among the YTH domain proteins, only YTHDC1 contains a selective binding pocket for the nucleotide preceding the m^6^A nucleotide [25]. Two pre-mRNA splicing factors SRSF3 and SRSF10 competitively bind to YTHDC1 [46]. It was proposed that YTHDC1 promotes exon inclusion by recruiting SRSF3 while blocking SRSF10 binding to target transcripts [46]. In this study, we find that YTHDC1 regulates mRNA splicing in oocytes. In addition, loss of YTHDC1 leads to formation of large novel cytoplasmic RNA-containing granules in the oocyte cytoplasm, which may contain aberrantly processed transcripts. Furthermore, the m^6^A-binding activity of YTHDC1 is required for rescue of alternative splicing defects in *Ythdc1*-deficient oocytes. Collectively, these *in vitro* and *in vivo* studies demonstrate the critical role of YTHDC1 in the regulation of alternative splicing, apparently in an m^6^A-dependent manner.

A number of studies show that m^6^A is a determinant of mRNA stability and turnover in the cytoplasm [23, 47, 76]. YTHDC1 facilitates nuclear export of m^6^A-containing mRNAs through its interaction with SRSF3 and thus regulates their cytoplasmic abundance [59]. YTHDC2 regulates the levels of m^6^A-containing transcripts in meiotic germ cells [32]. Binding by YTHDF2 causes redistribution of bound mRNAs to RNA degradation sites [23]. In addition, YTHDF2 regulates maternal mRNA clearance in both zebrafish and mouse [52, 53]. In contrast with the established role of m^6^A in mRNA turnover, the role of m^6^A in splicing has been a point of contention in the field. Some studies in ES cells conclude that m^6^A in nascent transcripts has a minor role in splicing, even though *Mettl3* inactivation in ES cells affects 3% of ~12,000 alternative cassette exons [47, 49]. It is possible that *Mettl3* inactivation may have more pronounced effect on splicing in differentiated cells. Indeed, *Mettl3* inactivation in male germ cells affects splicing [51]. The differentially spliced genes in *Ythdc1*-deficient oocytes significantly overlap with the differentially spliced genes in *Mettl3*-deficient testes (S8 Fig). Study of *Alkbh5*-deficient spermatogenic cells also supports a role of m^6^A in the regulation of splicing [74]. Therefore, the extent of effect on splicing by m^6^A most likely varies in different cell types and developmental stages.

## Materials and methods

### Ethics statement

Mice were maintained and used for experimentation according to the protocol approved by the Institutional Care and Use Committee of the University of Pennsylvania.

### Generation of polyclonal antibodies

The GST-YTHDC1 (aa 3–109) fusion protein (S2A Fig) was expressed in *E*. *coli* using the pGEX4T-1 vector and affinity purified with glutathione Sepharose 4B (GE Healthcare). Rabbits were immunized with recombinant protein, yielding antisera UP2410 and UP2411 (Cocalico Biologicals Inc.). For western blotting and immunofluorescence, antibodies were affinity purified against the GST fusion protein.

### Targeted inactivation of the *Ythdc1* gene

The *Ythdc1* targeting construct was designed to insert two tandem copies of *loxP-*flanked hygromycin phosphotransferase-thymidine kinase (HyTK) cassettes into *Ythdc1* intron 4, and a *loxP* site into intron 9 (S2B Fig). Genomic fragments were amplified from the *Ythdc1*-containing BAC clone RP24-567O8 by PCR with high-fidelity Taq DNA polymerase. The targeting construct was confirmed by sequencing. *Cla*I-linearized targeting construct was electroporated into V6.5 mouse embryonic stem (ES) cells, and ES cells were cultured in media containing 120 μg/ml hygromycin B. Of 368 hygromycin-resistant ES clones screened by long-range PCR, three clones were homologously targeted. Two positive clones (1A6 and 3D6) were expanded and electroporated with the *Cre*-expressing plasmid pOG231, followed by culture in media containing 2 μM ganciclovir. Ninety-six clones were screened for removal of the HyTK cassette and presence of *loxP* sites flanking *Ythdc1* exons 5–9 (S2B Fig), resulting in seven positive clones. Two (1A6H10 and 3D6G7) *Ythdc1*^*fl/+*^ ES clones were injected into blastocysts. The resulting chimeric mice transmitted the *Ythdc1* floxed allele through the germline.

Heterozygous (*Ythdc1*^*+/-*^) animals were produced by mating *Ythdc1*^fl/+^ with *Actb*-Cre mice [60]. Mice with conditional deletion of *Ythdc1* were obtained from the intercrosses of *Ythdc1*^fl/+^ with *Ddx4-*Cre or *Zp3-*Cre mice [61, 62]. The resulting *Ythdc1*^*fl/+*^ *Cre* males were crossed with *Ythdc1*^*fl/fl*^ females, yielding *Ythdc1*^*fl/-*^ *Cre* mice with germline-specific inactivation. Offspring were genotyped by PCR of genomic DNA with the following primers: wild-type (396 bp) and *Ythdc1* floxed allele (473 bp), CTTCCAGCAGGAATGAGTGC and GGCAATAAATAGCCCCAAAA; *Ythdc1*^-^ (deletion) (426 bp), GATATCTTCTCTGATTCATGCG and GGCAATAAATAGCCCCAAAA; *Ddx4*-Cre (240 bp), CACGTGCAGCCGTTTAAGCCGCGT and TTCCCATTCTAAACAACACCCTGAA; *Zp3*-Cre (220 bp), CCCAGATTCTGATCGTTGGT and CAGGTTCTTGCGAACCTCAT.

### Collection and culture of mouse oocytes, eggs and embryos

Full-grown, germinal vesicle (GV)-intact oocytes, metaphase II (MII) eggs, fertilized eggs and preimplantation embryos were collected as previously described [77, 78]. GV oocytes were cultured in Chatot-Ziomek-Brinster (CZB) medium [79] containing 2.5 μM milrinone (Sigma, St. Louis, MO, USA) to inhibit GV breakdown [80]; MII eggs were cultured in CZB medium and fertilized eggs/embryos cultured in KSOM [81].

GV oocytes were collected by poking of ovaries or enzymatic digestion. For enzymatic digestion, ovaries were dissected out and placed in Ca^2+^-Mg^2+^-free CZB medium containing 1 mg/ml collagenase (#LS004196, Worthington Biochemical Corp) and 0.2 mg/ml DNase I (Sigma #DN-25) in 35 mm petri dish. Each ovary was chopped into 4–5 pieces. Enzymatic digestion was carried out at 37°C for 40 min. Ovaries were pipetted up and down several times using a P1000 pipette to facilitate cell dissociation. Oocytes free of follicle cells were transferred and washed with three drops of CZB medium before further analysis.

### Western blotting and immunocytochemistry

Equal numbers of GV oocytes, metaphase I (MI) eggs, MII eggs, fertilized eggs and embryos were lysed in 2xSDS loading buffer (Sigma). Lysates were separated by 10% SDS-PAGE gel electrophoresis and proteins transferred to PVDF membrane (Amersham). For western blot analysis of adult mouse tissues, tissue samples were collected from 8-week-old adult mice and 20 μg of protein lysate per tissue analyzed per lane. The following antibodies/antisera were used for western blotting: rabbit anti-YTHDC1 affinity-purified antibody (this study); mouse anti-TUBB antibody (T4026, Sigma), mouse monoclonal ACTB (Clone AC-15, Sigma-Aldrich). Immuno-detection was performed using horseradish peroxidase-conjugated secondary antibodies and ECL prime reagents (Amersham) according to the manufacturer’s instructions.

For immunofluorescence, oocyte, egg or embryo samples were fixed in 2.5% paraformaldehyde for 40 min at room temperature. Cells were permeabilized for 15 min in PBS containing 0.2% Triton X-100, blocked in PBS containing 0.2% IgG-free BSA and 0.01% Tween-20 for 30 min (blocking solution), and then incubated with the rabbit anti-YTHDC1 affinity-purified antibody for 1 h at room temperature. After four 15-min washes in blocking buffer, samples were incubated for 1 h with appropriate Cy5-conjugated secondary antibody (Jackson ImmunoResearch). After three additional 15-min washes in blocking buffer, the samples were mounted in Vectashield mounting solution with Sytox green (Vector Laboratories). Images were captured by a Leica TCS SP laser-scanning confocal microscope.

Immunofluorescence analysis in testis was performed as previously described [82]. Briefly, adult or neonatal testes were fixed in 4% formaldehyde for 3–4 h and processed for sectioning in a cryostat. Testicular sections were immunostained with anti-YTHDC1 and anti-SP10 antibodies [83]. FITC- or Texas red-conjugated secondary antibodies were used. Slides were mounted in VectaShield solution with DAPI (Vector Laboratories). Images were captured with an ORCA digital camera (Hamamatsu Photonics) on a Leica DM5500B microscope.

### Whole-transcriptome RNA-seq analysis

Oocytes were collected from ovaries of 6-week-old wild-type or *Ythdc1*^fl/-^ *Ddx4*-Cre females by needle poking. Oocytes from *Ythdc1*^fl/-^ *Ddx4*-Cre females with gross abnormal morphology were excluded from studies. Total RNA was extracted from 25 oocytes per library using PicoPure RNA isolation kit with on-column genomic DNA digestion according to the manufacturer’s instruction (Thermo Fisher Scientific). As a normalization control, each sample was spiked in with 0.2 pg synthesized Renilla luciferase mRNA before extraction. RNA-seq libraries were constructed by using Ovation RNA-seq system V2 (NuGEN) followed by Ovation Ultralow Library system (DR Multiplex System, NuGEN). Reverse transcription of total RNA was primed with a pool of primers that hybridize either to the 5’ portion of the poly(A) sequence or randomly across the transcript. Per genotype, three biological replicate libraries were constructed. RNA-seq libraries were pooled and sequenced by three 150-bp paired-end runs on mid-output flow cells on the NextSeq 550 system (Illumina) (S1 Table). RNA-seq data are available under the NCBI/SRA number: SRP116737.

### Differential expression analysis

Oocyte RNA-seq data were mapped using the RNA-Seq aligner STAR. The STAR genome was generated using the mouse mm10 genome assembly (Genome Reference GRCm38). STAR was run with the parameter—clip3pAdapterSeq GATCGGAAGAGCACACGTCTGAACTCCAGTCAC. The SAM files from STAR were converted to BAM format using samtools view, reads were sorted by name using samtools sort, and separate lanes were merged into one file using samtools merge [84]. The number of reads in each genomic feature was quantified with HTSeq using the intersection-strict overlap setting [85]. Differential abundance between *Ythdc1*^fl/-^ and *Ythdc1*-deficient oocytes was then analyzed using the R package DESeq2 on the HTSeq count files with default settings and an FDR cutoff of 0.01 [86].

### Gene ontology (GO) analysis

Gene Ontology analysis was performed using the bioinformatics analysis resource database DAVID 6.8 [87]. Separate lists of differentially expressed up-regulated and down-regulated genes (all, FDR < 0.01, Fold change ≥ 2, and mean expression ≥100) were uploaded, with the Genbank_accession identifier selected and mus_musculus as the specified organism. A custom background list was supplied consisting of all genes with at least one read observed in any genotype or replicate in our RNA-seq libraries.

### Bioinformatic analysis of local splicing variants (LSVs) by MAJIQ

Analysis of units of LSVs between *Ythdc1*^fl/-^ and *Ythdc1*-deficient oocytes was performed using the MAJIQ software package [63]. MAJIQ v0.9.2 was run using the GRCm38 mm10 reference genome. Default settings were used for quantifying LSVs in the oocyte RNA-seq data and for the ΔPSI analysis. Please note that MAJIQ was not designed to identify alternative polyadenylation from RNA-seq data [63].

### Bioinformatic analysis of alternative polyadenylation by ROAR

Alternative polyadenylation (APA) analysis was performed with the ROAR Bioconductor package in R [67]. The package’s general workflow was followed. GV oocyte RNA-seq reads were mapped to the mm9 (NCBI37) genome using the RNA STAR aligner. ROAR was run using an annotation database of polyadenylation sites from the PolyADB version 2 [88]. The ratio of shorter to longer isoforms referred to as m/M ratio was computed for each sample using the counts of mapped reads and the lengths of the transcript’s PRE and POST portions as defined using a multiple APA annotation file. The ratio of the m/M_(WT)_ to the m/M_(KO)_ yielded the ratio of a ratio (ROAR) values, which were used to identify shifts in polyadenylation site usage. 3’ UTR lengthening or shortening was called when a Fisher test for all sample pairings returned nominal *p*-values < 0.05. We analyzed the RNA-seq data from the SRSF3 and SRSF7 knockdown experiments by Muller-McNicoll *et al*. [73] using the ROAR algorithm and reached the same conclusions on opposing changes on 3’ UTR length, validating the ROAR algorithm.

### Validation of differentially expressed genes by quantitative real-time PCR

Oocytes were collected from 6-week-old wild-type or *Ythdc1*^fl/-^ *Ddx4*-Cre or *Ythdc1*^fl/-^ *Zp3*-Cre females by needle poking of ovaries. Total RNA was extracted from oocytes using the PicoPure RNA Isolation Kit with on-column genomic DNA digestion (Thermo Fisher Scientific) and reverse transcribed by Superscript II reverse transcriptase (Invitrogen) using random hexamers. The resulting cDNA was quantified by real-time PCR on an ABI Prism 7000 thermocycler (Applied Biosystems) using Power SYBR Green Master Mix (Thermo Fisher Scientific). The following gene transcripts were tested: *Psat1*, *Grhl3*, *Cnnm1*, *Nupr*, *Zfp711*, *Lrp1b*, *Rgn*, *Trps1*, *Tnip3*, *Piwil1* (Oligonucleotide primer sequences in *S5 Table*). PCR parameters: 95˚C, 15 sec; 60˚C, 60 sec; 40 cycles. Each sample was analyzed in duplicates. Quantification was normalized to the endogenous *Actb* using the comparative Ct method (ABI Prism 7700 Sequence Detection System, Applied Biosystems).

### Validation of local splicing variants and APA

For validation of local splicing variants, each PCR assay was optimized individually (S5 Table). The PCR cycles varied for each LSV, depending on transcript abundance. For APA validation, two pairs of PCR primers were designed: PRE and POST (before and after the polyadenylation site) (S5 Table). All assays used an amount of cDNA equivalent to one oocyte per PCR reaction. RT-PCR band quantification was performed using ImageJ.

### Histological analysis

Testes and ovaries were prepared for histological analysis by fixation in Bouin’s solution (Sigma Aldrich), followed by serial dehydration and paraffin infiltration and embedding. Serial sections were cut at 8 μm thickness, adhered to glass slides, and dried overnight. Slides were de-paraffinized with xylene and re-hydrated. Slides were then stained with hematoxylin, rinsed, and exposed to 0.1 M ammonia before staining with eosin. The slides were then dehydrated and mounted with Permount mounting media (Fisher Scientific). Images were taken on a DM5500B microscopy platform with a DFC450 camera (Leica Microsystems).

### DNA constructs and mutagenesis

Wild-type mouse *Ythdc1* coding region was amplified from bulk mouse testis cDNA samples by PCR. The double mutation (W377A, W428A) was introduced by PCR-based mutagenesis by mutating codons 377 (TGG) 428 (TGG) to 377 (GCG) 428 (GCG), resulting in W377A W428A amino acid changes. The entire coding region was cloned into the pcDNA3.1 vector for in vitro transcription.

### In vitro transcription

Plasmids pcDNA3.1-wt-*Ythdc1* and pcDNA3.1-*Ythdc1*- W377A W428A were verified by sequencing and linearized before in vitro transcription. Capped mRNAs were made by in vitro transcription with T7 mScript mRNA production System (CellSCRIPT) according to the manufacturer’s instructions. Following in vitro transcription, template DNAs were digested by adding RNase-free DNase, and synthesized mRNA was purified by MEGAclear kit (Ambion). A single mRNA band of the expected size was observed for each RNA sample on a 1% formaldehyde denaturing gel. Synthesized RNA was stored in aliquots at -80°C.

### Oocyte microinjection and LSV rescue assay

GV oocytes were collected from postnatal day 12 *Ythdc1*-deficient ovaries by enzymatic digestion (collagenase). Oocytes were microinjected with approximately 5 pl of wild-type or mutant *Ythdc1* mRNA in water as previously described [89]. PND12 wild-type oocytes were mock injected with water as controls. Following microinjection, oocytes were returned to CZB medium with 2.5 μM milrinone and cultured overnight, followed by RNA extraction and reverse transcription. For LSV rescue experiments, each PCR assay was performed using an amount of cDNA equivalent to 0.5 oocyte. PCR bands were quantified using the Image J software. The rescue experiments were performed two times.

### Transfection and co-immunoprecipitation

The FLAG-YTHDC1 expression construct was made by cloning the full-length mouse *Ythdc1* coding sequence into a pcDNA6 vector containing a previously inserted 3xFLAG sequence 5’ of the cloning site. V5 tagged constructs for *Srsf7*, *Cstf1*, *Cstf2*, *Cpsf6*, and *Nudt21* were all produced by subcloning RT-PCR products amplified from bulk mouse testis cDNAs using the pcDNA 3.1/V5-His TOPO TA Expression Kit (Invitrogen, K4800). *Srsf3* cDNA was cloned into a pcDNA6 vector containing a Myc tag 5’ of the cloning site to express Myc-SRSF3. The HEK 293T cells were cultured in Dulbecco’s Modified Eagle Medium (DMEM) supplemented with 10% fetal bovine serum (FBS), 1% penicillin/streptomycin, and 1% L-glutamine in a 37°C humidified incubator at 5% CO_2_. Cells were co-transfected using Lipofectamine 2000 (Invitrogen) and cultured in Opti-mem media for 36 h. Transfected cells were harvested with RIPA buffer (10 mM Tris-HCl, pH 8, 1 mM EDTA, 0.5 mM EGTA, 1% Triton X-100, 0.1% SDS, 140 mM NaCl, 0.1% sodium deoxycholate) supplemented with 1 mM phenylmethylsulfonyl fluoride (PMSF). Cells were lysed by Dounce homogenization and solubilized by rocking at 4°C for 30 min. Following centrifugation at 16,100 x g for 25 min, lysate supernatants were incubated with 1 mg/ml RNase A at room temperature for 30 min. Lysates were cleared by centrifugation at 16,100 x g for 20 min and incubated with Protein G agarose beads (Invitrogen, 15920010) for 1 h. After pre-clearing, either anti-FLAG (F-3165, Sigma), anti-V5 (R96025, ThermoFisher), or anti-Myc (631206, Clontech) was incubated with cell lysates rotating overnight at 4°C. Equilibrated Protein G agarose beads were added to the lysates and incubated for 1 h. Immunoprecipitated protein complexes were washed three times with RIPA buffer supplemented with PMSF. Beads and respective input lysates were boiled with 2x SDS sample buffer for 5 min prior to SDS-PAGE and immunoblotting to nitrocellulose membranes.

## Supporting information

S1 Fig Nuclear localization of YTHDC1 in transcriptionally active male germ cells: Spermatogonia, spermatocytes, and round spermatids. (A) Frozen testicular sections from 8-week-old wild-type males were immunostained with anti-YTHDC1 and anti-SP10 antibodies. SP10 (also called ACRV1) is a component of the acrosome and thus used for seminiferous tubule staging [83]. DNA was stained with DAPI. Tubules at stages V, IX, and XII are shown. Scale bar, 50 μm. (B) Summary of YTHDC1 protein expression during spermatogenesis. The diagram of spermatogenesis was re-drawn as previously illustrated [90]. Expression of YTHDC1 protein is shown in green. Stages (I–XII) of spermatogenesis are shown. Spg, spermatogonia; PL, pre-leptotene; L, leptotene; Z, zygotene; P, pachytene; D, diplotene; M, metaphase spermatocyte; RS, round spermatid; and ES, elongating spermatid.
(TIF)

S2 Fig Conditional inactivation of the *Ythdc1* gene. (A) The only known motif in YTHDC1 is the YTH domain. The antibody was raised against the N-terminal region encompassing amino acids (aa) 3–109. Mouse YTHDC1 protein reference sequence: NP_808348.2. (B) Diagram of wild-type and targeted *Ythdc1* alleles. Mouse *Ythdc1* maps to Chromosome 5 and consists of 17 exons. Targeted deletion of exons 5–9 (aa 296–452) results in a frame shift in the transcribed mRNA and removes the YTH domain. (C) Ubiquitous inactivation of *Ythdc1* is embryonic lethal. Timed matings of *Ythdc1*^fl/-^ mice were set up, and embryos/pups collected and genotyped at the time points shown. Numbers in brackets marked with asterisks indicate the number of resorptions found.
(TIF)

S3 Fig Absence of YTHDC1 protein in male germ cells from neonatal *Ythdc1*^fl/-^ *Ddx4*-Cre testes. Frozen testicular sections from neonatal wild-type and *Ythdc1*^fl/-^ *Ddx4*-Cre males were immunostained with anti-YTHDC1 antibody. Nuclear DNA was stained with DAPI. Gonocytes (also called prospermatogonia) are indicated by white arrowheads. YTHDC1 is nuclear in wild-type gonocytes but absent in *Ythdc1*^fl/-^ *Ddx4*-Cre gonocytes. Scale bar, 25 μm.
(TIF)

S4 Fig Absence of YTHDC1 protein in *Ythdc1*^fl/-^ *Ddx4*-Cre oocytes. Oocytes were collected from 6-week-old mice. (A) Western blot analysis of oocytes from wild-type and *Ythdc1* cKO (*Ythdc1*^fl/-^ *Ddx4*-Cre) females. TUBB (β-tubulin) served as a loading control. (B) YTHDC1 immunostaining of wild-type and *Ythdc1* cKO (*Ythdc1*^fl/-^ *Ddx4*-Cre) oocytes. Nuclei/nuclear DNA and cytoplasmic RNA granules are marked by arrowheads (blue) and arrows (white), respectively. Sytox green stains both DNA and RNA. Please note that all the Sytox green signals in the wild type oocyte were from nuclear DNA staining.
(TIF)

S5 Fig Dysregulated transcriptome in *Ythdc1*-deficient oocytes. (A) Scatter plot of transcript profiling between wild-type and *Ythdc1*^fl/-^ *Ddx4*-Cre oocytes from 6-week-old females. FDR cutoff: 0.01. The list of differentially expressed transcripts is shown in S2 Table. (B) Validation of 10 differentially expressed genes by real-time PCR. Real-time PCR was performed in duplicates. The average and range are shown. (C) GO term enrichment in up-regulated and down-regulated genes in *Ythdc1*-deficient oocytes. Differentially expressed genes with FDR < 0.01, Fold change ≥ 2, and mean expression ≥ 100 were included in the GO analysis.
(TIF)

S6 Fig GO term enrichment in MAJIQ-called genes with LSVs between wild-type and *Ythdc1*-deficient oocytes. (TIF)

S7 Fig PCR validation of LSVs affecting internal exons in *Ythdc1*-deficient oocytes. Oocytes were collected from 6-week-old *Ythdc1*^fl/+^ and *Ythdc1*^fl/-^ *Zp3*-Cre females. Exons are represented as rectangles but not in scale. Skipped or retained exons are shown in red. Triangles denote the positions of PCR primers.
(TIF)

S8 Fig Significant overlap of differentially spliced genes in *Ythdc1*-deficient oocytes and *Mettl3* knockout testes. The RNA-seq data from control and *Mettl3* knockout postnatal day 12 testes from the previous Xu *et al* study [51] were re-analyzed by MAJIQ. Statistics was performed by hypergeometric enrichment tests.
(TIF)

S1 Table Sequence reads of six oocyte RNA-seq libraries (3 wild-type and 3 *Ythdc1* cKO). (XLSX)

S2 Table Differentially expressed genes between wild-type and *Ythdc1*-deficient oocytes from 6-week-old females. FDR cutoff of 0.01 was used.
(XLSX)

S3 Table MAJIQ-identified local splicing variants (LSVs) between wild-type and *Ythdc1*-deficient oocytes from 6-week-old females. (XLSX)

S4 Table ROAR output of alternative polyadenylation events between wild-type and *Ythdc1*-deficient oocytes. (XLSX)

S5 Table Oligos for qPCR, LSV validation, and APA validation. (XLSX)
