# Supplementary figures and images for: Nuclear m6A reader YTHDC1 regulates alternative polyadenylation and splicing during mouse oocyte development
Source: PLoS Genet. 2018 May 25;14(5):e1007412. doi: 10.1371/journal.pgen.1007412 (PMC5991768; doi:10.1371/journal.pgen.1007412)

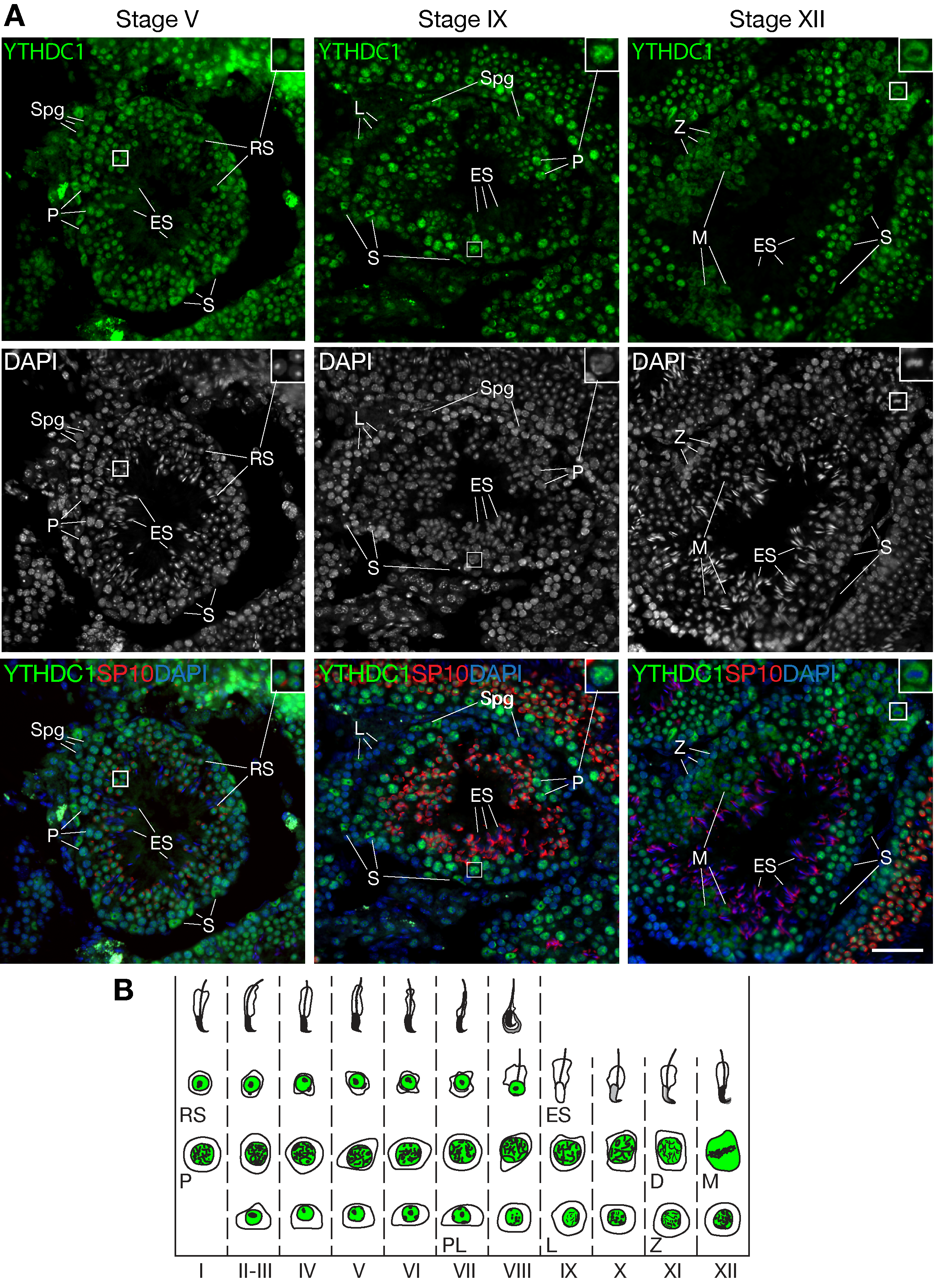

Supplement: S1 Fig — (A) Frozen testicular sections from 8-week-old wild-type males were immunostained with anti-YTHDC1 and anti-SP10 antibodies. SP10 (also called ACRV1) is a component of the acrosome and thus used for seminiferous tubule staging [83]. DNA was stained with DAPI. Tubules at stages V, IX, and XII are shown. Scale bar, 50 μm. (B) Summary of YTHDC1 protein expression during spermatogenesis. The diagram of spermatogenesis was re-drawn as previously illustrated [90]. Expression of YTHDC1 protein is shown in green. Stages (I–XII) of spermatogenesis are shown. Spg, spermatogonia; PL, pre-leptotene; L, leptotene; Z, zygotene; P, pachytene; D, diplotene; M, metaphase spermatocyte; RS, round spermatid; and ES, elongating spermatid. (TIF) [file pgen.1007412.s001.tif]

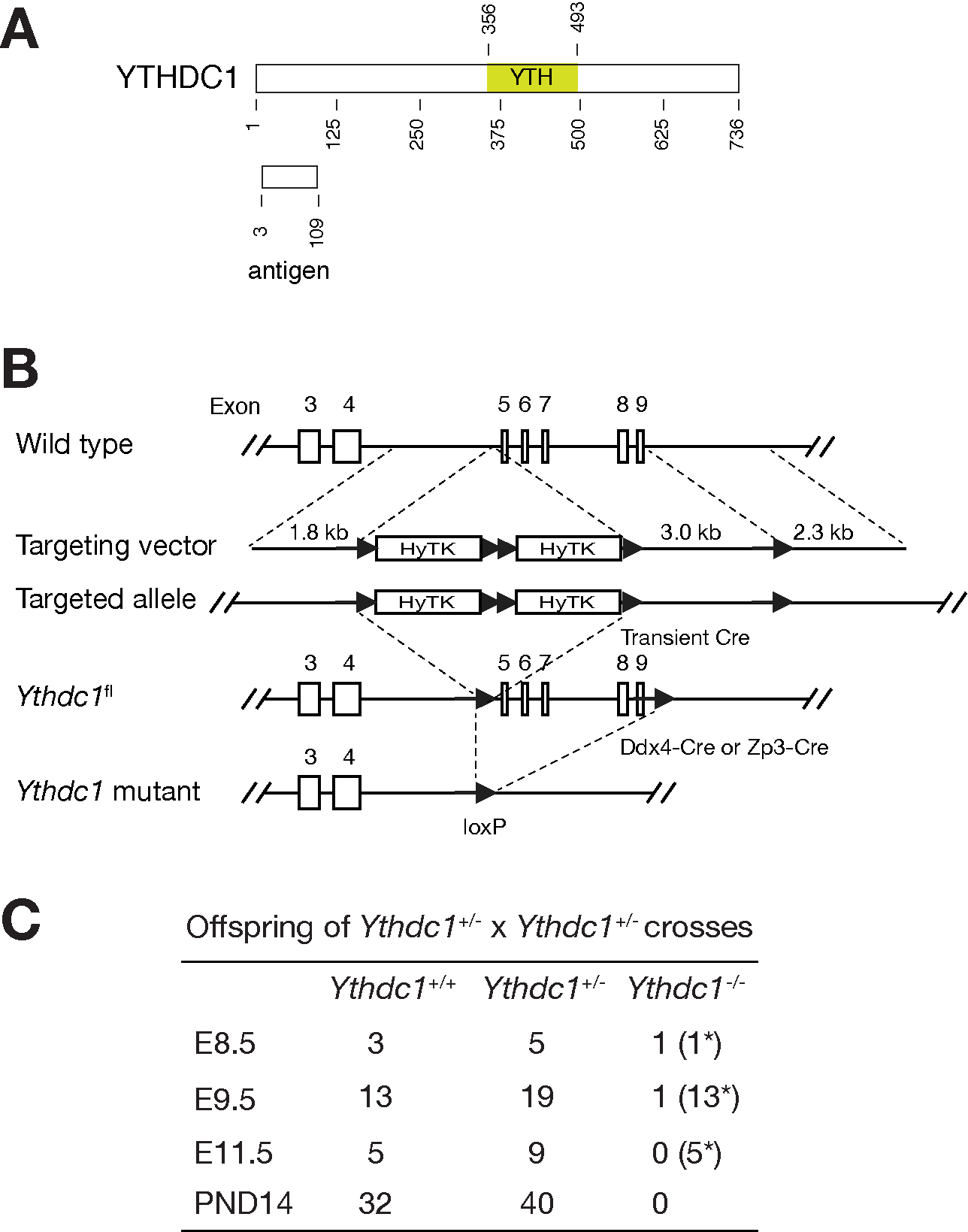

Supplement: S2 Fig — (A) The only known motif in YTHDC1 is the YTH domain. The antibody was raised against the N-terminal region encompassing amino acids (aa) 3–109. Mouse YTHDC1 protein reference sequence: NP_808348.2. (B) Diagram of wild-type and targeted Ythdc1 alleles. Mouse Ythdc1 maps to Chromosome 5 and consists of 17 exons. Targeted deletion of exons 5–9 (aa 296–452) results in a frame shift in the transcribed mRNA and removes the YTH domain. (C) Ubiquitous inactivation of Ythdc1 is embryonic lethal. Timed matings of Ythdc1fl/- mice were set up, and embryos/pups collected and genotyped at the time points shown. Numbers in brackets marked with asterisks indicate the number of resorptions found. (TIF) [file pgen.1007412.s002.tif]

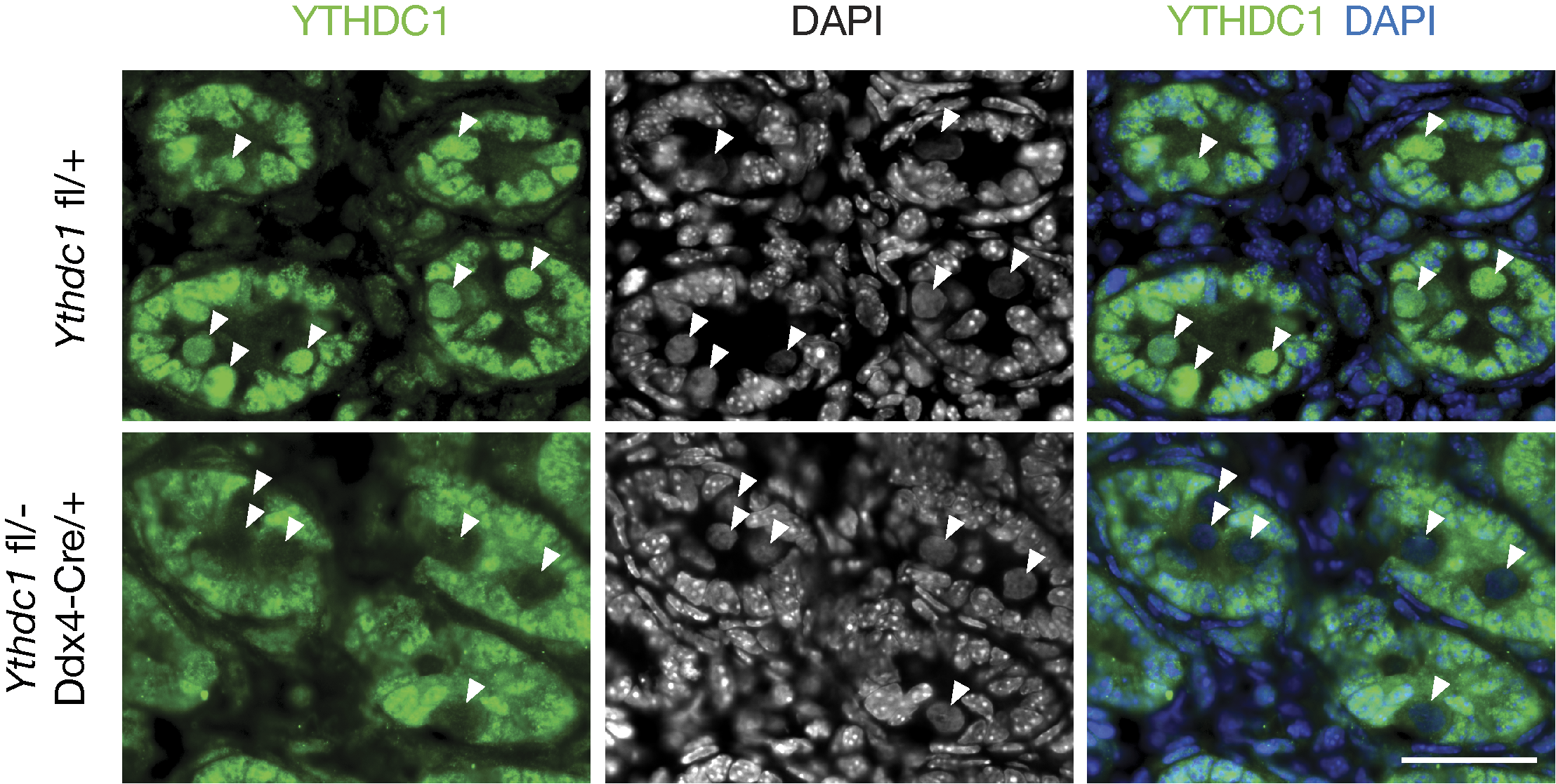

Supplement: S3 Fig — Frozen testicular sections from neonatal wild-type and Ythdc1fl/- Ddx4-Cre males were immunostained with anti-YTHDC1 antibody. Nuclear DNA was stained with DAPI. Gonocytes (also called prospermatogonia) are indicated by white arrowheads. YTHDC1 is nuclear in wild-type gonocytes but absent in Ythdc1fl/- Ddx4-Cre gonocytes. Scale bar, 25 μm. (TIF) [file pgen.1007412.s003.tif]

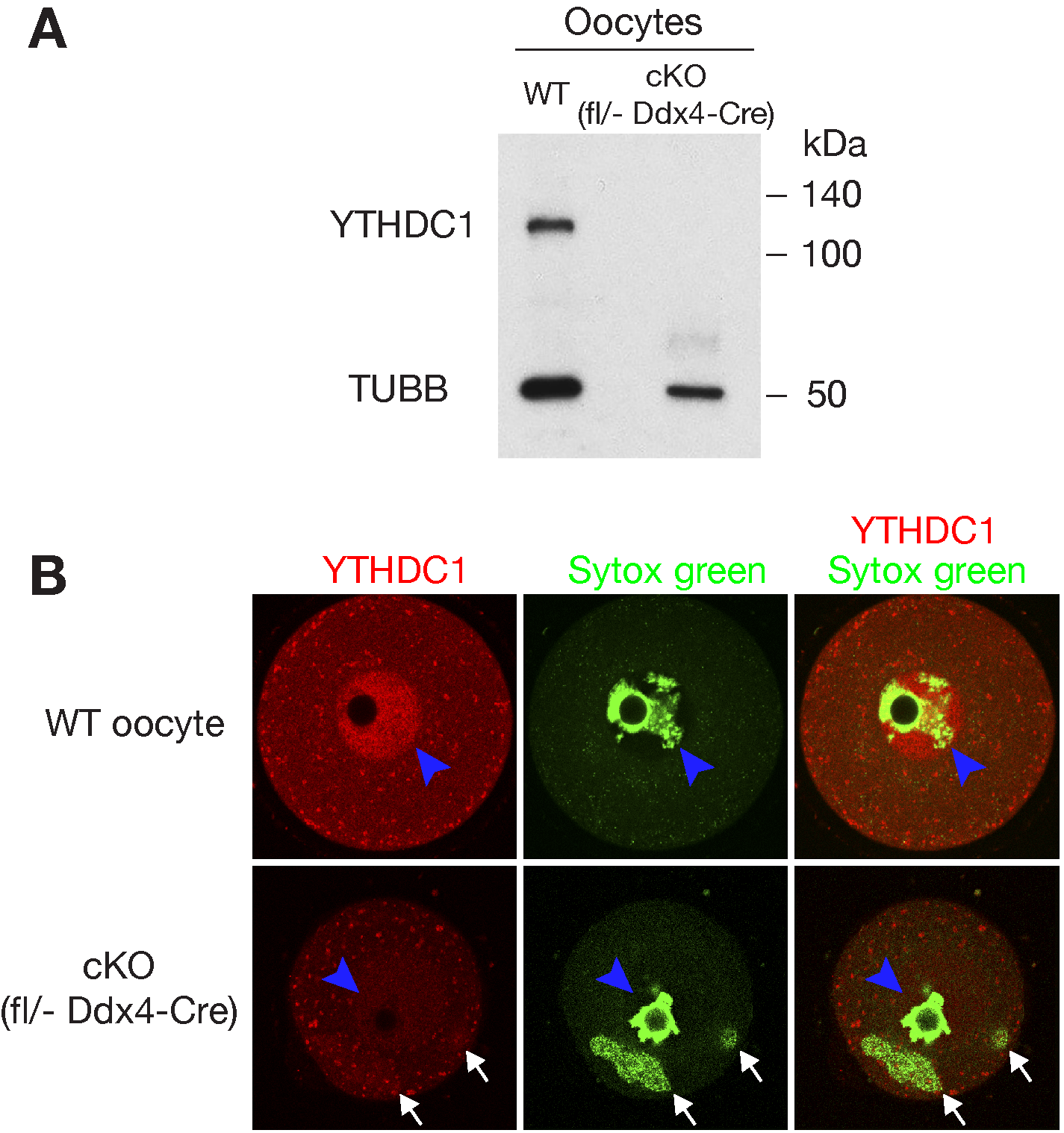

Supplement: S4 Fig — Oocytes were collected from 6-week-old mice. (A) Western blot analysis of oocytes from wild-type and Ythdc1 cKO (Ythdc1fl/- Ddx4-Cre) females. TUBB (β-tubulin) served as a loading control. (B) YTHDC1 immunostaining of wild-type and Ythdc1 cKO (Ythdc1fl/- Ddx4-Cre) oocytes. Nuclei/nuclear DNA and cytoplasmic RNA granules are marked by arrowheads (blue) and arrows (white), respectively. Sytox green stains both DNA and RNA. Please note that all the Sytox green signals in the wild type oocyte were from nuclear DNA staining. (TIF) [file pgen.1007412.s004.tif]

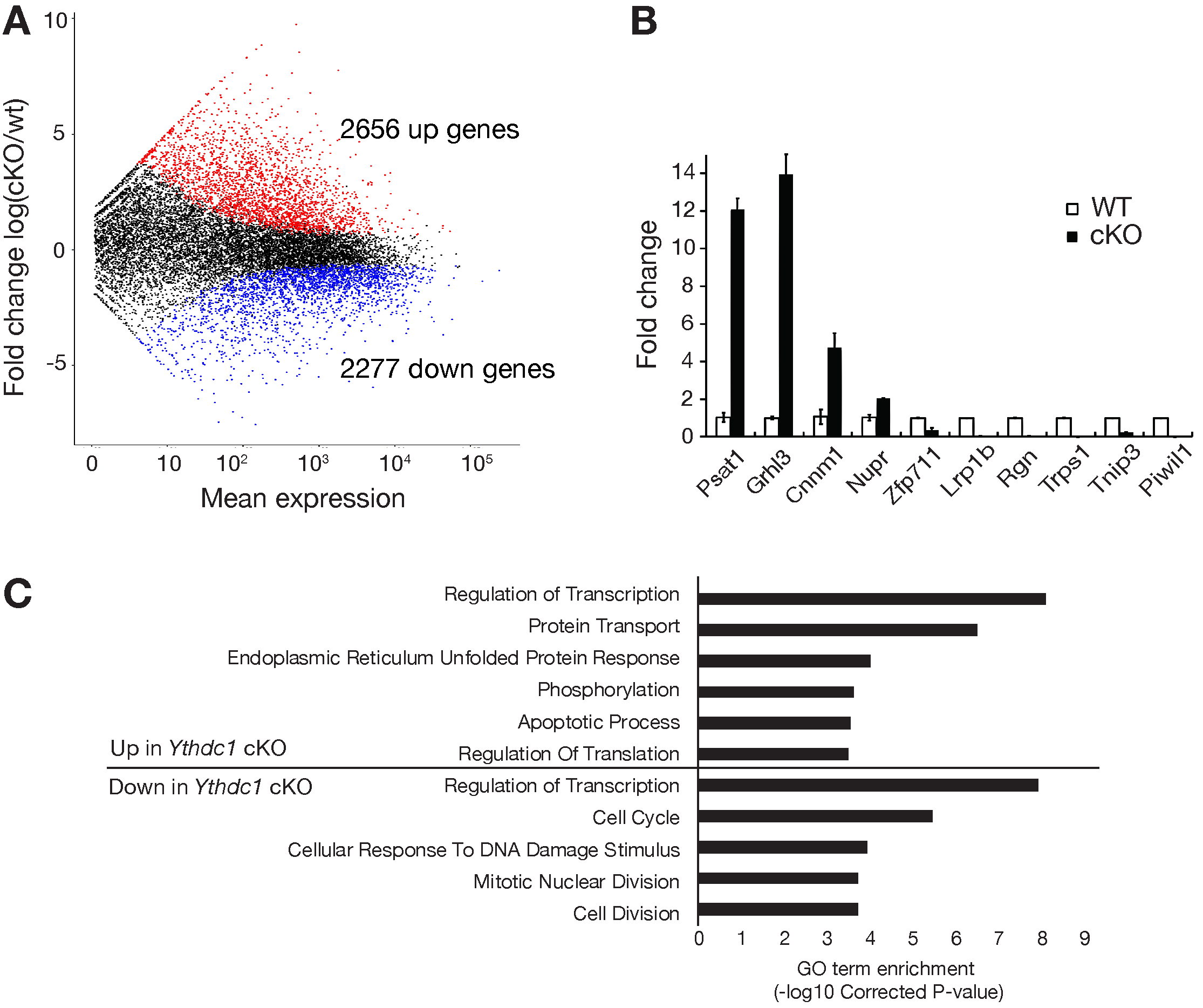

Supplement: S5 Fig — (A) Scatter plot of transcript profiling between wild-type and Ythdc1fl/- Ddx4-Cre oocytes from 6-week-old females. FDR cutoff: 0.01. The list of differentially expressed transcripts is shown in S2 Table. (B) Validation of 10 differentially expressed genes by real-time PCR. Real-time PCR was performed in duplicates. The average and range are shown. (C) GO term enrichment in up-regulated and down-regulated genes in Ythdc1-deficient oocytes. Differentially expressed genes with FDR < 0.01, Fold change ≥ 2, and mean expression ≥ 100 were included in the GO analysis. (TIF) [file pgen.1007412.s005.tif]

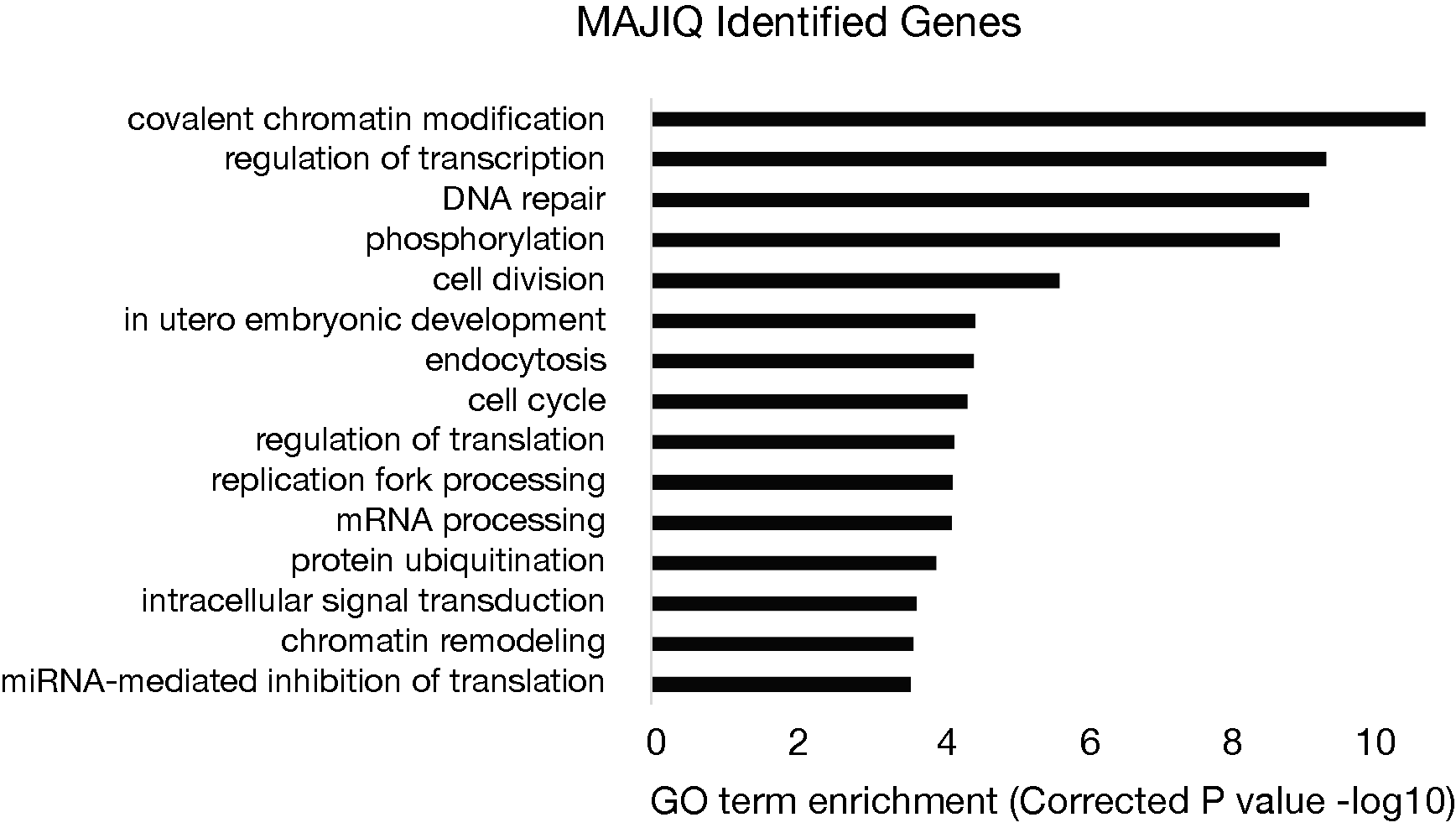

Supplement: S6 Fig — (TIF) [file pgen.1007412.s006.tif]

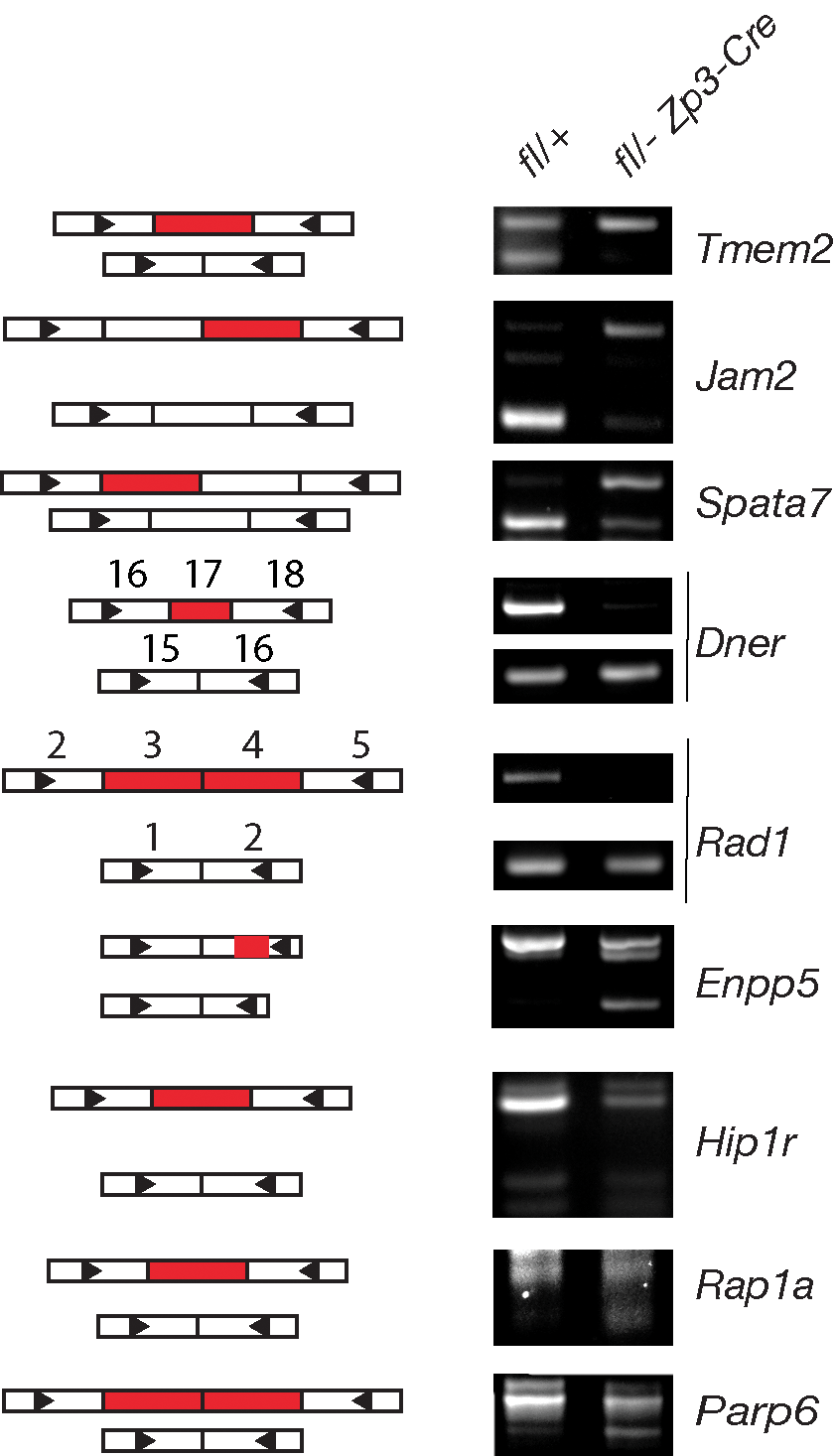

Supplement: S7 Fig — Oocytes were collected from 6-week-old Ythdc1fl/+ and Ythdc1fl/- Zp3-Cre females. Exons are represented as rectangles but not in scale. Skipped or retained exons are shown in red. Triangles denote the positions of PCR primers. (TIF) [file pgen.1007412.s007.tif]

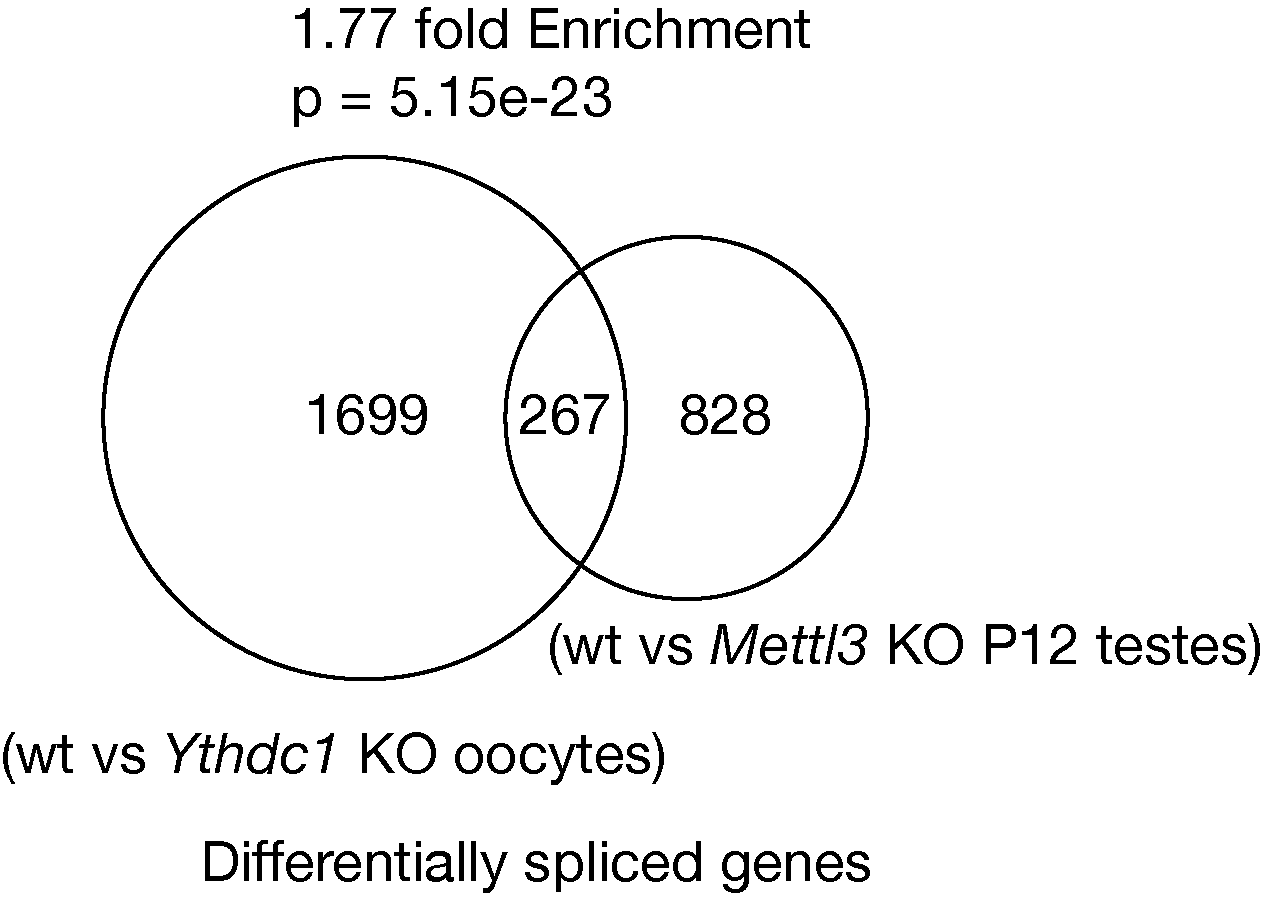

Supplement: S8 Fig — The RNA-seq data from control and Mettl3 knockout postnatal day 12 testes from the previous Xu et al study [51] were re-analyzed by MAJIQ. Statistics was performed by hypergeometric enrichment tests. (TIF) [file pgen.1007412.s008.tif]
